# LPC-engineered liposomes activate transcytosis for paclitaxel brain delivery and potentiation of TTFields/TMZ via TALECT regimen in glioblastoma

**DOI:** 10.7150/thno.122658

**Published:** 2026-01-01

**Authors:** Qi Zhan, Kaikai Yi, Shixue Yang, Biao Hong, Dongyuan Su, Jixing Zhao, Qixue Wang, Xiaoteng Cui, Yanping Huang, Yaqing Ding, Chunchao Cheng, Jiasheng Ju, Chunsheng Kang

**Affiliations:** 1 Laboratory of Neuro-Oncology, Tianjin Neurological Institute, Tianjin Medical University General Hospital, Key Laboratory of Post-Neuro Injury Neuro-Repair and Regeneration in Central Nervous System, Ministry of Education and Tianjin City, 154 Anshan Road, Tianjin 300052, China.; 2 Department of Neuro-Oncology and Neurosurgery, Tianjin Medical University Cancer Institute and Hospital, National Clinical Research Center for Cancer, Key Laboratory of Cancer Prevention and Therapy of Tianjin, Tianjin's Clinical Research Center for Cancer, West Huan-Hu Road, Tianjin 300060, China.

**Keywords:** glioblastoma, TTFields, paclitaxel, blood-brain barrier, LPC liposomes

## Abstract

**Rationale:** Tumor Treating Fields (TTFields) in combination with temozolomide (TMZ) provides significant survival benefits in glioblastoma (GBM), but prolonged daily use of TTFields compromises patient compliance and cost-effectiveness. Paclitaxel (PTX) has potential synergistic effects with TTFields, but its efficacy is limited by the blood-brain barrier (BBB). The BBB penetration efficiency of receptor-mediated nanocarriers is limited by low endocytosis activity and lysosomal entrapment in brain endothelial cells.

**Methods:** To overcome this limitation, we developed lysophosphatidylcholine (LPC)-engineered liposomes (LPC-Lipo) to activate brain endothelial transcytosis for BBB crossing PTX delivery and established a paclitaxel liposomes-electric fields-TMZ (TALECT) regimen for TTFields-based GBM therapy. The transcytosis behavior and mechanism of LPC-Lipo were investigated in both* in vitro* and *in vivo* BBB models. *In vivo* PTX delivery efficiency of LPC-Lipo and the therapeutic efficacy of TALECT regimen were evaluated in a GBM patient-derived xenograft model.

**Results:** Leveraging our findings that LPC can activate brain endothelial transcytosis, LPC-Lipo was developed and exhibited enhanced transcytosis efficiency through accelerated endocytosis and directional trafficking to recycling endosomes (bypassing lysosomes) mediated by p62/SQSTM1, thereby achieving efficient BBB penetration. PTX-loaded LPC-Lipo achieved effective GBM suppression, and this efficacy was replicated in the clinical-grade Lipusu^®^ upon LPC engineering. Integrating optimized PTX delivery with the TTFields/TMZ combination established the TALECT regimen. TALECT therapy achieved potent therapy outcomes while reducing daily TTFields exposure by 75%. This efficacy stems from PTX's dual sensitization: (1) to TTFields via microtubule disruption-induced mitotic arrest, and (2) to TMZ by inhibiting DNA damage repair.

**Conclusions:** This work presents a novel transcytosis-activating platform for BBB penetration and proposes the clinically translatable TALECT regimen, advancing the cost-effectiveness of TTFields-based GBM therapy.

## Introduction

Glioblastoma (GBM) remains the most lethal primary brain tumor, with a median survival of 14.6 months despite multimodal therapy 1, 2. Tumor Treating Fields (TTFields), the first new GBM therapy incorporated into clinical guidelines in over a decade, achieved a median overall survival exceeding 20 months for the first time when combined with temozolomide (TMZ) 3, 4. TTFields deliver alternating electric fields via transducer arrays placed on the scalp. However, the current regimen requires prolonged daily use (≥ 18 h), imposing significant compliance barrier and prohibitive costs that limit patient accessibility 5, 6. This limitation in cost-effectiveness necessitates synergistic strategies to sensitize GBM to TTFields/TMZ therapy, thereby shortening the required TTFields exposure duration.

TTFields exert antitumor effects primarily by disrupting microtubule polarized alignment and dynamics, inducing mitosis arrest 7. This mechanism identifies microtubule-targeting agents as prime candidates for combination therapy. Paclitaxel (PTX), a classic microtubule-stabilizing agent, induces mitosis arrest by inhibiting microtubule depolymerization. This mechanistic synergy motivated our hypothesis that PTX could enhance GBM's sensitivity to TTFields. Supporting this, a recent ovarian cancer clinical trial demonstrated the safety and efficacy of PTX combined with standard TTFields, confirming this combination's clinical feasibility 8. However, applying this regimen to GBM faces a major obstacle: PTX, despite potent *in vitro* efficacy against GBM (IC_50_ ~1,400-fold lower than TMZ) 9, fails to achieve therapeutic efficacy *in vivo* due to the blood-brain barrier (BBB). The BBB establishes a highly restrictive barrier through its tightly connected endothelial cell layer 10. Conventional PTX formulations (e.g., Taxol^®^, Abraxane^®^, and Lipusu^®^), while improving solubility, pharmacokinetics, and systemic safety, fail to achieve therapeutic brain concentrations due to low BBB transport efficiency 9. Thus, innovative BBB-penetrant PTX formulations are critically needed to unlock its combinatorial potential with TTFields in GBM.

Emerging nanotechnologies, including peptide-drug conjugates, polymer nanoparticles, and liposomal formulations, provide a promising platform for brain-targeted delivery 11, 12. These BBB-crossing strategies primarily rely on active transport mediated by ligand-receptor interactions, where ligands conjugated to the nanoparticle surface to target specific receptors highly expressed on the BBB 13-15. Despite progress, such designs often focus solely on receptor binding and overlook the transcytosis process, which is a dynamic sequence requiring endocytosis, vesicular trafficking, and exocytosis to complete BBB penetration 16-18. Notably, two fundamental barriers limit transcytosis efficacy: (1) Brain endothelial cells (bECs) exhibit inherently low endocytic activity, potentially impeding transcytosis initiation 19, 20, and (2) Ligand-modified nanoparticles post-endocytosis are frequently trafficked to lysosomes for degradation rather than to recycling endosome for exocytosis, significantly reducing transcytosis efficiency 17, 21, 22. Together, these limitations restrict the BBB-penetration efficiency of nanoparticles, resulting in insufficient drug delivery. To overcome these barriers, next-generation nanoparticles must be rationally engineered to actively activate endothelial transcytosis, thereby enhancing PTX delivery for GBM therapy.

**Figure 1:**
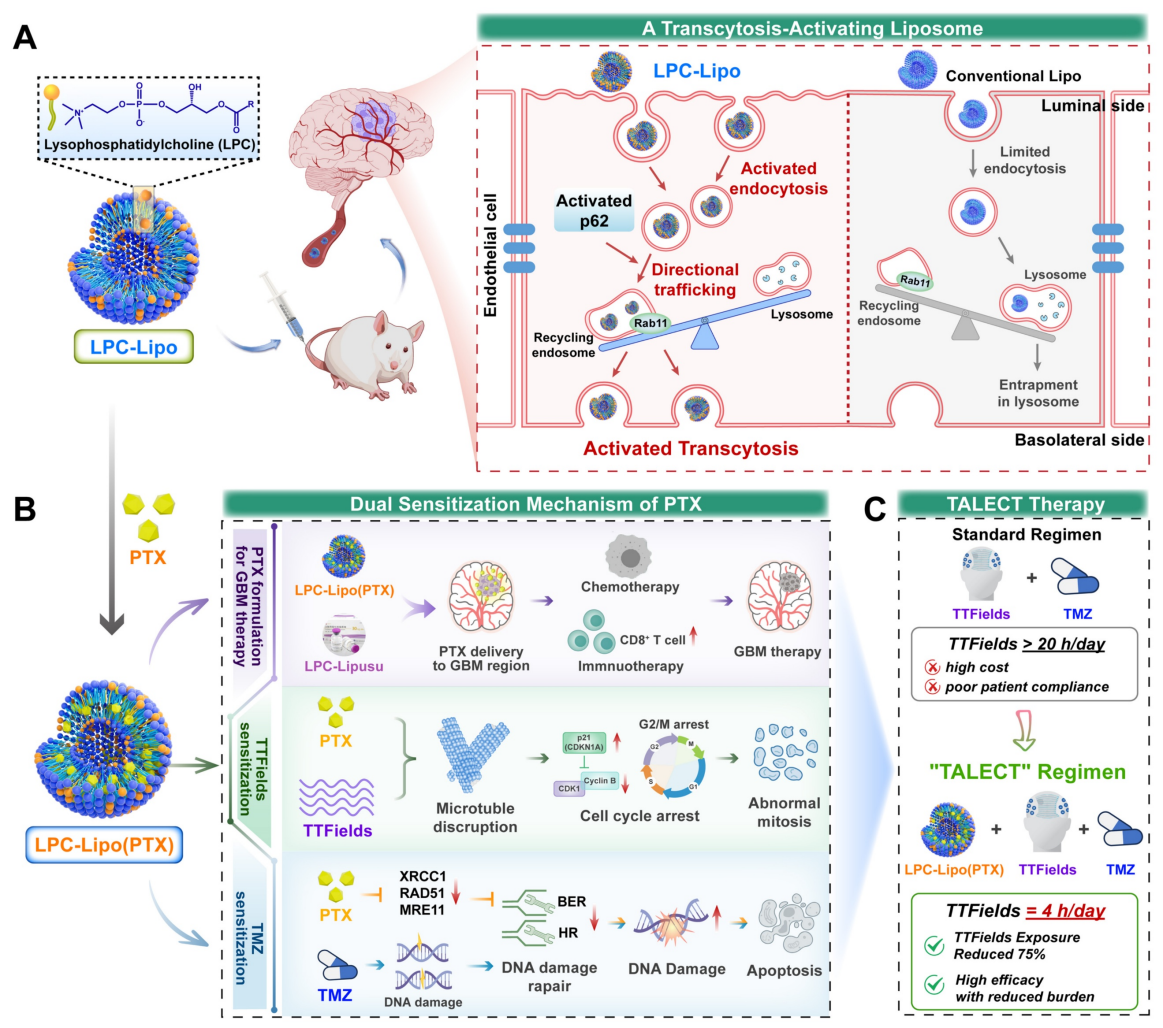
Schematic illustration of a transcytosis-activating PTX liposomes for shortened TTFields duration in GBM therapy. (A) Schematic illustration of LPC liposomes activating brain endothelial transcytosis through enhanced endocytosis and p62-mediated directional trafficking. (B) Schematic illustration for LPC-Lipo-mediated PTX brain delivery and the PTX's dual sensitization mechanisms to TTFields/TMZ regimen. (C) Novel TALECT therapy combining LPC-Lipo(PTX) with a TTFields regimen featuring a 75% reduction in daily exposure duration.

In this study, we developed an innovative lysophosphatidylcholine (LPC) liposomes to activate brain endothelial transcytosis for BBB crossing PTX delivery. We explored its potential as a novel PTX formulation in GBM therapy, and established a pacli**ta**xel **l**iposomes-el**ec**tric fields-**T**MZ (TALECT) therapy to reduce TTFields exposure duration (**Figure 1**). Phospholipids, as key membrane components, regulate vesicle formation and trafficking 23. Our previous work revealed that LPC, a single-tailed phospholipid, modulates membrane-associated processes, particularly endocytosis and exocytosis 24, 25. We thus hypothesized that LPC can function as a transcytosis-regulating molecule. Herein, we demonstrate that LPC enhances the endocytosis and transcytosis activities of bECs by increasing membrane plasticity. Building on this, we engineer LPC liposomes (LPC-Lipo) by incorporating LPC into conventional phosphatidylcholine (PC) liposomes (Lipo). LPC-Lipo activates transcytosis through enhanced endocytosis and redirecting endocytic vesicles toward Rab11^+^ recycling endosomal pathways (away from lysosomal degradation) via a p62-mediated mechanism, enabling effective BBB penetration (**Figure 1A**).

We further applied this liposomal platform to formulate PTX for GBM treatment. Our novel PTX formulation, LPC-Lipo(PTX), achieves higher intratumoral drug concentration than conventional PTX liposomes (Lipo(PTX)), leading to superior chemotherapeutic and immunotherapeutic efficacy. This enhancement is also observed in LPC-engineered Lipusu^®^. Crucially, combining LPC-Lipo(PTX) with TTFields/TMZ therapy reveals a dual sensitization mechanism: (1) Sensitization to TTFields via microtubule disruption and mitotic arrest, and (2) Sensitization to TMZ through DNA damage repair inhibition (**Figure 1B**). This TALECT therapy shortens daily TTFields exposure requirements by 75% while enhancing therapeutic efficacy, significantly improving cost-effectiveness of TTFields regimen (**Figure 1C**). Collectively, this work presents a transcytosis-activating nanoplatform that not only offers a novel PTX formulation for GBM treatment but also provides new insights and solutions for optimizing the clinical cost-effectiveness of TTFields-based GBM therapy.

## Materials and Methods

### Materials and Cell Culture

Lysophosphatidylcholine (LPC, #L1381 and #L4129), phosphatidylcholine (PC, #P3556), and cholesterol (#C8667) were purchased from Sigma-Aldrich (USA). Paclitaxel (PTX, #S1150) and temozolomide (TMZ, #S1237) were purchased from Selleck (China). Lipusu^®^ was obtained from Luye Pharma Group (China). Cy 5 and Cy 5.5 dyes were purchased from Yuanye BioTechnology Co., Ltd (China). Murine brain endothelial cell line bEnd.3, human GBM cell line U87, and mouse GBM cell line CT2A were cultured in DMEM supplemented with 10% fetal bovine serum (FBS). Patient-derived GBM cells (TBD0220) were cultured in DMEM/F12 supplemented with 10% FBS. Lentiviruses expressing mouse p62 shRNA (sequence: GAGGTTGACATTGATGTGGAA) were synthesized by GenePharma (China).

### Fluorescence Recovery after Photobleaching (FRAP) Membrane Fluidity Assay

Control and LPC-treated bEnd.3 cells were incubated in confocal dishes and stained with 1 μg/mL DiI solution (Sigma #42364), followed by three washes with culture medium. Cells were observed using a 100x oil immersion objective on a confocal laser scanning microscope (CLSM). A small, distinct area of membrane was photobleached using laser pulses; a 30-s scan achieved complete or near-complete photobleaching. Fluorescence intensity was recorded every 10 s after photobleaching. After background normalization, fluorescence intensity was plotted over time to generate recovery curves.

### Scanning Ion Conductance Microscopy (SICM)

The bEnd.3 cells were seeded at 50% confluence, washed three times with L15 medium, and imaged by SICM (Ionscope Ltd., UK). Scanning a 75 × 75 μm area at 128 × 128-pixel resolution required ~20 min. Control cells underwent continuous scanning. For LPC-treated cells, an initial scan was acquired, followed by gradual addition of LPC solution to the culture medium and subsequent continuous scanning. All experiments were performed at room temperature. 3D image reconstruction and topographical analysis used SICMImage Viewer 2010.

### Preparation and Characterization of Liposomes

LPC-incorporated liposomes (LPC-Lipo) were prepared via the thin-film hydration method with PC, cholesterol, and LPC (at 5%, 10%, and 20% w/w of total lipids). Briefly, the lipid components were dissolved in chloroform, and the solvent was removed by rotary evaporation under vacuum to form a thin lipid film. The film was further dried under vacuum overnight and subsequently hydrated with distilled water. The resulting suspension was ultrasonicated in an ice-water bath and then extruded sequentially through 0.2 μm polycarbonate membranes (Mini-extruder, Avanti Polar Lipids, USA) to obtain liposomes with a uniform size. Conventional liposomes (Lipo) without LPC served as the control. For drug-loaded formulations (Lipo(PTX) and LPC-Lipo(PTX)), PTX was added to the initial lipid solution prior to film formation. The liposomes were purified by dialysis to remove unencapsulated PTX. For fluorescence labeling, DiI or DiR dyes were incorporated during the lipid film preparation step.

The particle size distribution, polydispersity index (PDI) and zeta potential were measured at 25°C by dynamic light scattering (NanoBrook 90plus PALS, Brookhaven Instruments, USA). To determine the drug-loading capacity (DLC) and drug encapsulation efficiency (EE), the purified Lipo(PTX) and LPC-Lipo(PTX) were treated with 1% Triton X-100 to disrupt the lipid membrane and release the encapsulated PTX. The amount of PTX was quantified via high-performance liquid chromatography (HPLC, Waters Alliance System) using a C18 column with acetonitrile/water (75:25, v/v) mobile phase at a flow rate of 1.0 mL/min, and detection was carried out at 227 nm. The DLC and EE were calculated as follows:

DLC (%) = [*W*_encapsulated / (*W*_encapsulated* + W_*lipid)] × 100%

EE (%) = (*W*_encapsulated* / W*_input) × 100%

where *W*_encapsulated is the amount of PTX encapsulated in the liposomes, *W_*lipid is the amount of total lipids, and *W*_input is the initial amount of PTX used.

### *In Vitro* BBB Transcytosis Assay

Transcytosis efficiency was evaluated using an *in vitro* BBB model established in a 24-well Transwell cell culture system (Corning, USA). The bEnd.3 cells were seeded onto the upper chamber and cultured until monolayer integrity was confirmed by stable trans-endothelial electrical resistance (TEER) measurements. For Tfn transcytosis studies: Monolayers were pretreated with 50 μM LPC for 30 min, followed by apical application of FITC-labeled Tfn (100 μg/mL). Basolateral medium was collected after 12 h incubation, with fluorescence intensity quantified using a TECAN microplate reader (ex/em 494/518 nm). For liposomes transcytosis studies, DiI-labeled Lipo or LPC-Lipo were applied apically at 50 μg/mL. Basolateral medium was collected at 0.5, 1, 2, 4, 6, 12 and 24 h intervals, with DiI fluorescence quantified (ex/em 549/565 nm). Transport efficiency (%) of both studies was calculated as (Fluorescence _basolateral_ / Fluorescence _apical(initial)_) × 100. Monolayer integrity was verified pre-/post-assay via TEER maintenance and 70 kDa FITC-dextran (Invitrogen) exclusion (< 1% permeability). Parallel experiments employed shp62-transduced bEnd.3 monolayers. For glioma studies, TBD0220 cells in basolateral chambers were analyzed by flow cytometry after 12-h LPC-Lipo exposure.

### Establishment of orthotopic patient-derived xenograft (PDX) model

All animal procedures were approved by the Animal Ethics Welfare Committee (AEWC) of Tianjin Medical University General Hospital (Protocol No. IRB2023-DW-136). Intracranial PDX models were established in 4-6-week-old female BALB/c nude mice. The GBM patient-derived TBD0220 cells were implanted into brain parenchyma of nude mice under the guidance of a stereotactic instrument at coordinates relative to bregma: 2.0 mm posterior, 2.0 mm lateral, 3.0 mm ventral.

### *In Vivo* Anti-tumor Activity of LPC-Lipo(PTX)

Seven days after intracranial implantation of TBD0220 cells, tumor-bearing nude mice were randomly divided into four groups (n = 5) and treated intravenously via tail vein with Saline, PTX, Lipo(PTX), and LPC-Lipo(PTX) (both 8 mg/kg PTX-equivalent). Treatments were administered every three days for five times in total. Intracranial tumor growth was monitored by bioluminescence imaging (IVIS Spectrum, PerkinElmer) on days 7, 14, and 21. On day 21, the mice were sacrificed; and their brain tissues were harvested, fixed in 4% PFA, and paraffin-embedded for hematoxylin and eosin (H&E) staining. Kaplan-Meier survival curves were generated in independent animal cohorts.

### Preparation of LPC-Lipusu and *In Vivo* Therapeutic Evaluation

Lipusu^®^ was incubated with LPC (10 μM) in PBS buffer for 30 min at 37 °C under gentle rotation. LPC molecules were incorporated into Lipusu^®^ through hydrophobic interactions between their acyl tail and the liposomal lipid layer, forming LPC-Lipusu formulations. Unbound LPC was removed by ultracentrifugation (100,000 × g, 45 min, 4 °C) and washed twice with PBS. Therapeutic efficacy was evaluated in orthotopic PDX model. Mice received intravenous injections of Saline, native Lipusu^®^, and LPC-Lipusu (8 mg/kg PTX-equivalent). Treatments were administered every 3 days for five total doses. Tumor progression was monitored by bioluminescence imaging. Survival analysis was performed in separate cohorts (n = 6). Histopathological evaluation was conducted on day 21 as previously described.

### *In Vivo* Tumor Treating Fields (TTFields) Experiments

The efficacy of TALECT therapy was evaluated using a PDX model established as above mentioned. Mice were randomly assigned to four experimental groups: (1) Saline (control), (2) TMZ, (3) TTFields + TMZ, and (4) LPC-Lipo(PTX) + TTFields + TMZ. TMZ was administered *via* oral gavage at a dose of 5 mg/kg on a schedule of 5 consecutive days of treatment followed by a 2-day rest period, repeated for two weeks. LPC-Lipo(PTX) was administered intravenously at a dose of 5 mg/kg PTX-equivalent every three days, for a total of five doses. For TTFields application, electrode arrays coupled with conductive gel were positioned over the following four regions: one at the midpoint between the ipsilateral ear and eye (applied bilaterally), one midway between the ears, and one midway between the eyes. Electrodes and connecting wires were securely fixed to prevent dislodgement, and mice were individually housed throughout the TTFields application period. Electrode positioning and integrity were monitored daily and replaced as needed. TTFields (200 kHz) were delivered with group-specific durations: the TTFields + TMZ group received 20 h/day exposure, while the LPC-Lipo(PTX) + TTFields + TMZ group received 4 h/day exposure. TTFields therapy was administered every other day. Tumor growth was monitored via bioluminescence imaging, and animal survival was recorded. Post-treatment, brain tissues were harvested for subsequent histological analysis, including H&E staining and Ki67 immunohistochemistry analysis.

### Statistics

Statistical analyses were performed with GraphPad Prism 8.0 Software. All data are presented by mean ± standard deviation (s.d.). The paired Student's t-test was used to compare experimental groups and control groups. For multiple experimental groups, one-way or two-way ANOVA was used. Significance was defined as *p* < 0.05 and ns means no significant differences.

### Other Methods

For materials and methods related to transmission electron microscopy (TEM), molecular dynamics (MD) simulation, liquid chromatography-tandem mass spectrometry (LC-MS/MS) proteomics analysis, cellular endocytosis assay, BBB penetration imaging, intracellular trafficking localization analysis, western blot (WB), intracranial PTX concentration assay, anti-tumor activity analysis in CT2A model, flow cytometry analysis of tumor-infiltrating T cells, *in vitro* TTFields assay, and DNA damage analysis see **Supplementary Material** for details.

## Results and Discussion

### LPC Enhances Endocytosis and Transcytosis Activities of bECs

**Figure 2:**
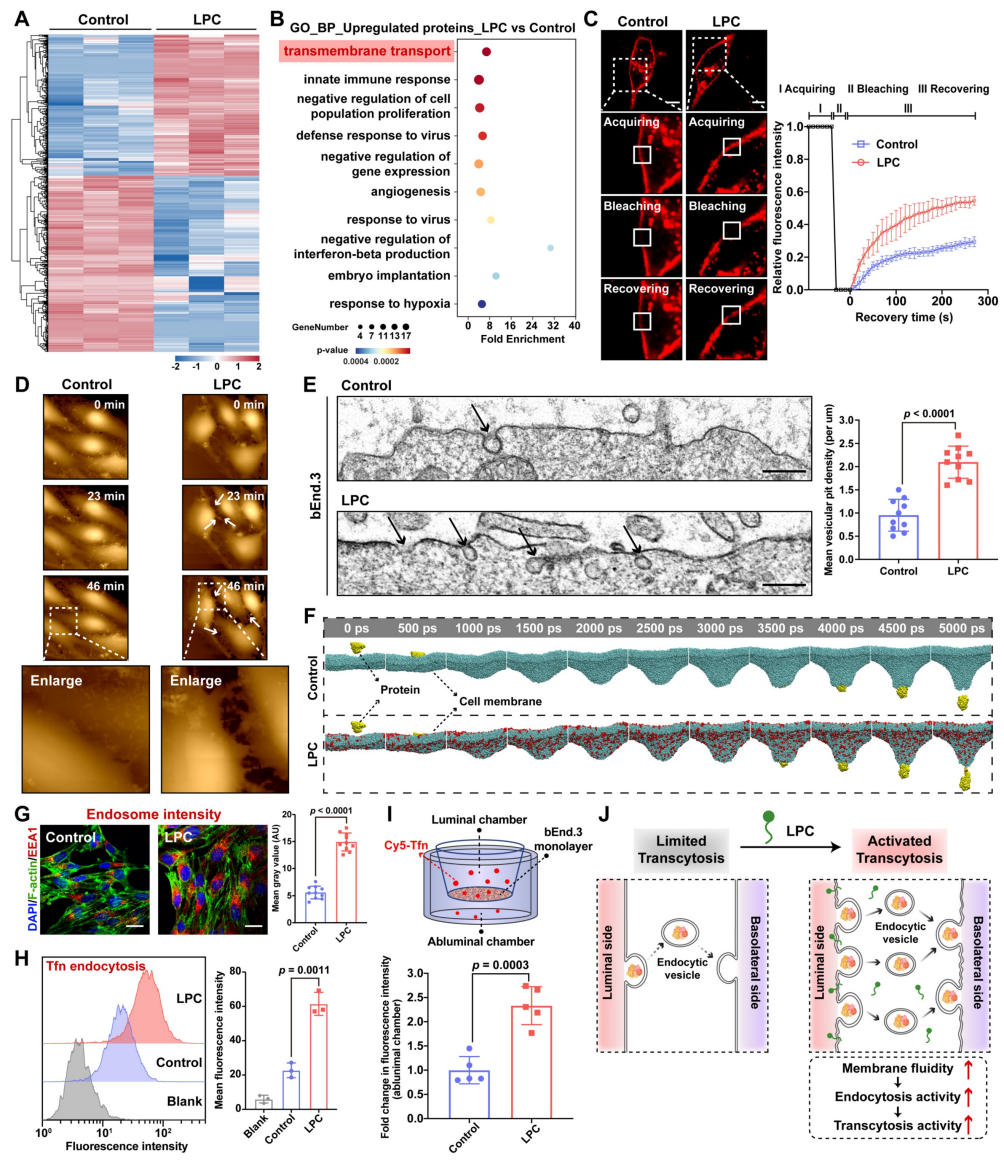
LPC stimulates endocytosis and transcytosis in brain endothelial cells. (A) Heatmap of differentially expressed proteins in bEnd.3 cells following LPC treatment compared with control. (B) GO enrichment analysis of proteins upregulated by LPC treatment relative to control. (C) Confocal images and corresponding fluorescence recovery curve from FRAP analysis in LPC-treated bEnd.3 cells (n = 5). (D) SICM images of bEnd.3 cells after LPC addition, with the image taken at 0 min as baseline. (E) TEM images showing vesicular pits on the plasma membrane of LPC-treated bEnd.3 cells (n = 10). (F) MD simulation snapshots showing the pulling-induced penetration of a protein particle through the cell membrane. Membrane-incorporated LPC is colored red. (G) Fluorescence images and corresponding quantification showing the early endosome density (labeled with EEA1, red) in LPC-treated bEnd.3 cells (n = 10). (H) Flow cytometry analysis of Cy5-Tfn endocytosis in LPC-treated bEnd.3 cells (n = 3). (I) Transcytosis efficiency of Cy5-Tfn across the bEnd.3 monolayer in an *in vitro* BBB model under LPC treatment (n = 5). (J) Schematic illustration of LPC-enhanced endocytosis and transcytosis in bECs. Scale bar: 20 μm (C, G); 150 nm (E). Data are presented as mean ± s.d. Statistical significance was analyzed by unpaired two-tailed Student's *t*-test for E, G, and I and one-way ANOVA with Tukey's multiple comparisons test for H.

The transcytosis process across the BBB involves three sequential stages: (i) endocytosis of particles into bECs, (ii) transport of endocytic vesicles to recycling endosome, and (iii) exocytosis to the brain parenchyma (Figure S1A). LPC, a lysophospholipid characterized by a single fatty acyl chain, has previously been shown to regulate endocytosis and exocytosis in tumor cells. Here, we investigated whether LPC regulates brain endothelial transcytosis. Proteomic profiling of LPC-treated bEnd.3 cells identified 249 upregulated and 310 downregulated proteins (|Log_2_(fold change)| > 0.58, *p* < 0.05; FDR-adjusted) among 6,323 proteins compared to control (Figure S1B). All differentially expressed proteins are depicted in the heatmap (Figure 2A). Gene Ontology (GO) enrichment analysis of the upregulated proteins revealed significant enrichment in transmembrane transport pathways (Figure 2B), consistent with our hypothesis.

To experimentally validate LPC's regulatory role in transcytosis, we focused on membrane plasticity, which is essential for membrane invagination during vesicle formation in endocytosis and vesicle-membrane fusion during exocytosis 26. We characterized LPC-induced membrane perturbation in bEnd.3 cells. Fluorescence recovery after photobleaching (FRAP) analysis demonstrated a significantly enhanced membrane fluidity in LPC-treated cells, evidenced by a 90% increase in membrane fluorescence recovery kinetics at 100 s post-bleaching compared to control (Figure 2C). Furthermore, live-cell membrane monitoring using scanning ion conductance microscopy (SICM) revealed rapid membrane extension and deformation upon LPC administration, contrasting with stable membrane morphology in untreated cells (Figure 2D), indicating increased membrane flexibility. Enhanced membrane fluidity directly facilitates invagination formation. Transmission electron microscopy (TEM) analysis confirmed this, showing a 2.2-fold increase in the density of membrane micro-invaginations in LPC-treated cells (Figure 2E). Molecular dynamics (MD) simulations using a protein-mimetic particle further confirmed these findings. Simulation snapshots (Figure 2F) showed accelerated membrane invagination and penetration kinetics in the presence of LPC, with initial invagination occurring at 500 ps and membrane penetration achieved by 3500 ps. Throughout the simulation, the LPC system exhibited greater invagination distances at equivalent timepoints than the control system (Figure S1C). These results demonstrate that LPC enhances membrane plasticity, a critical biophysical driver of vesicle formation and transport during transcytosis.

We next assessed the effects of LPC on endocytosis and transcytosis in bEnd.3 cells. We first found that LPC treatment increased the fluorescence intensity of early endosomes labeled with Early Endosome Antigen 1 (EEA1) by 2.7-fold (Figure 2G). EEA1 is an established marker for early endosomes, and its elevated levels reflect enhanced endocytic activity. This finding was further supported by increased EEA1 protein levels (Figure S2A), confirming LPC's ability to promote endocytic vesicle formation. This effect depended on the source and concentration of LPC, as evaluated by EEA1 levels (Figure S2B). Comparative analysis indicated that LPC, particularly bovine brain-derived LPC containing unsaturated fatty acyl chains, was more effective than PC, which shares the same choline headgroup but possesses two hydrophobic tails. Moreover, this efficacy diminished at excessive concentrations. While the choline head group of LPC may contribute the recognition at the BBB similarly to its analogues 27, its functional superiority likely stems primarily from its single hydrophobic tail, which induces membrane perturbation to promote endocytosis 28, 29. However, excessive concentrations can lead to membrane lysis 30, reducing effectiveness. To evaluate LPC's impact on cargo transport, we employed transferrin (Tfn), a classic transcytosis molecule. Confocal laser scanning microscopy (CLSM) and flow cytometry (FC) analysis revealed that LPC significantly enhanced the endocytosis efficiency of Cy5-labelled Tfn in bEnd.3 cells (Figure 2H). This increase aligns with the observed promotion of endocytic vesicles (Figure S2C), indicating LPC-mediated activation of endothelial endocytic activity. Furthermore, using an *in vitro* BBB model, we detected a 2.3-fold increase in Cy5-Tfn fluorescence intensity within the basal chamber of LPC-treated bEnd.3 monolayer compared to control (Figure 2I), indicating enhanced endothelial transcytosis activity under LPC exposure. Notably, trans-endothelial electrical resistance (TEER) and paracellular permeability assays confirmed preserved endothelial integrity post-LPC treatment (Figure S2D-E). Together, these findings demonstrate that LPC regulates membrane plasticity and vesicle formation, thereby activating both endocytosis and transcytosis of bECs (Figure 2J). Given the inherently low endogenous transcytosis activity of bECs, which limits the transcytosis efficiency of nanoparticles across the BBB, LPC presents a promising strategy for designing nanocarriers capable of activating transcytosis for enhanced brain delivery.

### Development of LPC-Engineered Liposome for Enhanced BBB Penetration

**Figure 3:**
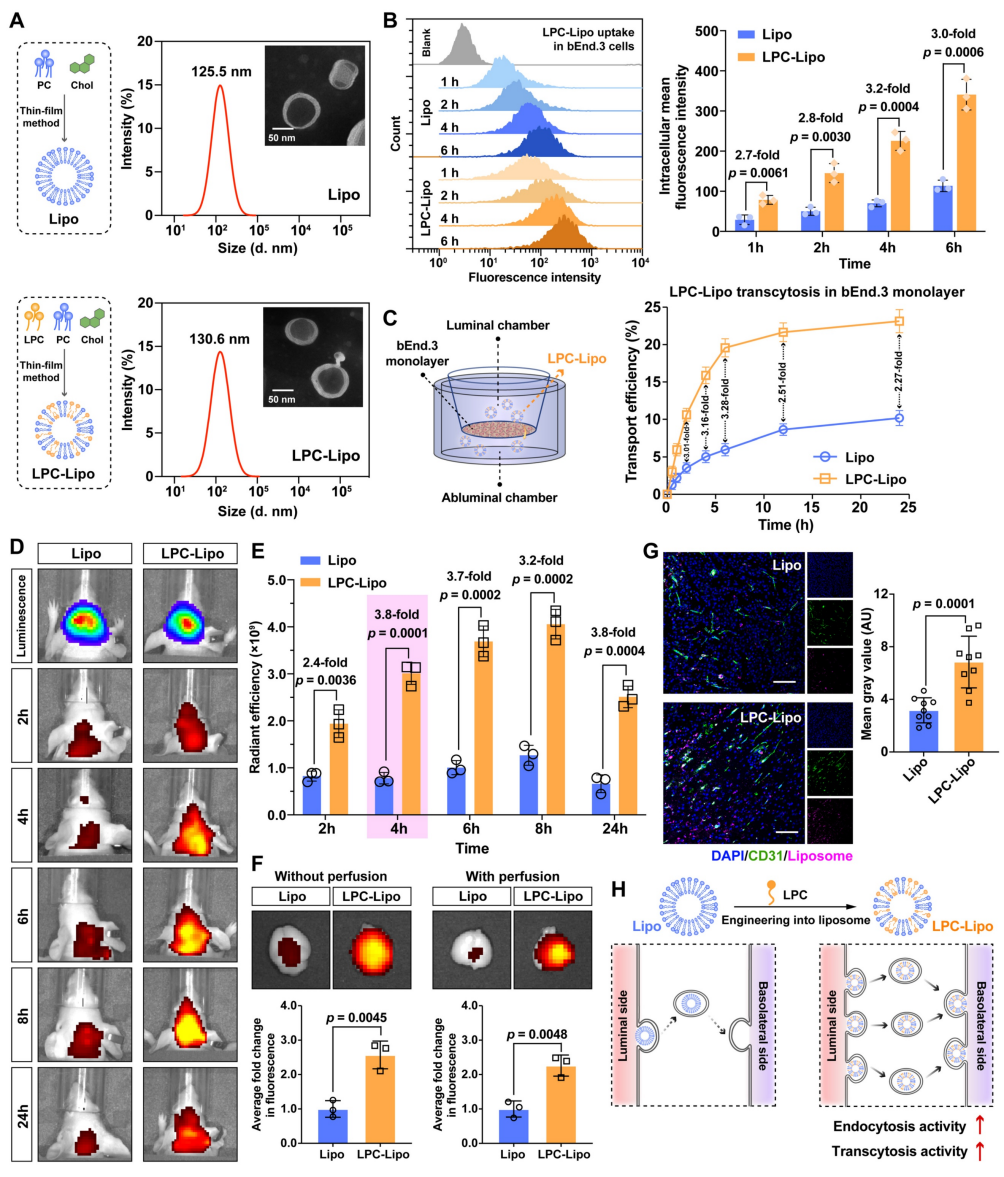
Characterization and BBB penetration performance of LPC-engineered liposomes (LPC-Lipo). (A) Representative TEM images (scale bar, 50 nm) and corresponding particle size distribution of conventional liposomes (Lipo) and LPC-Lipo. (B) Time-dependent endocytosis of Lipo and LPC-Lipo in bEnd.3 cells analyzed by flow cytometry (left) and quantified fluorescence intensity (right) (n = 3). (C) Cumulative transcytosis efficiency of Lipo and LPC-Lipo in an *in vitro* BBB model at indicated time points (n = 3). (D) *In vivo* real-time fluorescence tracking of DiR-labeled Lipo and LPC-Lipo in the brain region of orthotopic GBM (TBD0220) model mice. (E) Quantified fluorescence intensity in the brain region (n = 3). (F) *Ex vivo* fluorescence images and quantified fluorescence intensity of brains from mice injected with Lipo and LPC-Lipo, collected with or without perfusion (n = 3). (G) Confocal images of tumor region in brain slices showing CD31^+^ blood vessels (green) and DiR-labeled liposomes (pink), along with corresponding quantification of LPC-Lipo (n = 10). Scale bar: 100 μm. (H) Schematic illustration of LPC-enhanced liposomes transcytosis across the BBB. Data are presented as mean ± s.d. Statistical significance was analyzed by unpaired two-tailed Student's *t*-test for B, E, F, and G.

Liposomes constitute a well-established drug delivery platform and represent the most clinically approved and widely utilized nanocarrier system 31, 32. However, their efficacy in brain tumor therapy is constrained by inefficient BBB penetration. Based on our findings that LPC activates transcytosis and leveraging its structural compatibility with liposomes, we developed LPC-engineered liposomes to investigate their BBB-penetrating potential. LPC was incorporated into clinically approved standard liposomes composed of PC and cholesterol. Transmission electron microscopy revealed that both Lipo and LPC-Lipo formed monodisperse spherical vesicles with well-defined membrane boundaries (**Figure 3A**). Dynamic light scattering (DLS) showed that the hydrodynamic diameters of Lipo and LPC-Lipo were 125.5 nm and 130.6 nm, respectively, confirming that LPC incorporation did not compromise liposomal integrity. Further DLS analysis of LPC-Lipo with LPC contents of 5%, 10%, and 20% showed consistent hydrodynamic diameters (approximately 130 to 140 nm) and low PDI values (0.19 to 0.21), indicating a uniform and monodisperse distribution of all formulations (**Figure S3A**). However, when LPC content exceeded 20%, the liposome solution exhibited a heterogenous state with obvious phase separation, indicating an inability to form a stable, homogeneous dispersion (**Figure S3B**). This instability is likely attributable to the monoacyl chain structure of LPC, where excessive incorporation disrupts hydrophobic packing density and impairs bilayer stability. Zeta potential measurements indicated negative surface charges for all formulations, with values of -39.59 ± 3.20 mV (Lipo), -39.64 ± 1.06 mV (5% LPC-Lipo), -33.58 ± 2.94 mV (10% LPC-Lipo), and -31.97 ± 2.52 mV (20% LPC-Lipo) (**Figure S3C**). The negative charge of LPC-Lipo may contribute to serum stability via electrostatic repulsion. All formulations maintained excellent colloidal stability over 7 days at 4 °C, with no significant changes in particle size or zeta potential (**Figure S3D-E**), except for the 20% LPC-Lipo, which showed increases in both particle size and zeta potential by day 7, likely due to membrane instability from high LPC content.

We next assessed endocytosis and transcytosis of LPC-Lipo in bECs. Fluorescence imaging revealed colocalization of LPC-Lipo with EEA1^+^ endocytic vesicles in bEnd.3 cells. Dynasore (an endocytosis inhibitor) significantly reduced basolateral LPC-Lipo signals in an *in vitro* BBB model (**Figure S4A-B**), confirming that transcytosis initiates via endocytosis. Cytotoxicity assays confirmed the good biocompatibility of all LPC-Lipo formulations (5-20% LPC) in bEnd.3 cells (**Figure S4C**). We then systematically evaluated the functional performance of these formulations. Quantitative results showed that all LPC-Lipo formulations (containing 5-20% LPC) exhibited significantly enhanced endocytosis and transcytosis efficiency compared to conventional Lipo. Notably, the 10% LPC-Lipo formulation achieved the highest endocytosis and transcytosis activity (**Figure S4D-E**). Therefore, the 10% LPC-Lipo formulation was selected for all subsequent studies. Time-course analysis showed that LPC-Lipo achieved endothelial internalization levels equivalent to Lipo at 4 h within just 1 h, with a 3.2-fold efficiency enhancement at 4 h (**Figure 3B**), indicating that LPC accelerated the endocytosis of liposomes in bECs. In the *in vitro* BBB model, the transcytosis of LPC-Lipo was significantly enhanced, showing elevated fluorescence signals in the basal chamber as early as 1 h post-administration. Efficiency peaked at 6 h with a 3.3-fold enhancement over Lipo and stabilized at a 2.3-fold increase (**Figure 3C**). Particle size analysis confirmed intact LPC-Lipo in the basal chamber, while TEER measurements verified preserved bEnd.3 monolayers integrity (**Figure S5A-B**), confirming transcytosis without structural compromise. Furthermore, GBM cells cultured in the basal chamber exhibited significantly higher LPC-Lipo uptake compared to Lipo (**Figure S5C**). These results demonstrate that LPC incorporation enhances both endocytosis and transcytosis activities of liposomes in endothelial cells (**Figure 3H**).

We next evaluated BBB penetration using an orthotopic GBM patient-derived xenograft (PDX) model. Bioluminescence imaging delineated tumor regions, while fluorescence imaging tracked intracerebral distribution post-intravenous injection. LPC-Lipo exhibited robust fluorescence signals in brain within 2 h, persisting for 24 h with significantly higher accumulation than Lipo (**Figure 3D**). ROI analysis quantified a 3.8-fold greater cerebral fluorescence intensity for LPC-Lipo at 4 h, sustained through 24 h (**Figure 3E**). *Ex vivo* imaging confirmed stronger cerebral fluorescence in the LPC-Lipo group, particularly post-perfusion, with robust persistent LPC-Lipo signals but nearly undetectable Lipo signals (**Figure 3F**). Quantification revealed a 2.3-fold increase in brain accumulation of LPC-Lipo, indicating its potentiated BBB penetration efficiency. Histology showed extensive LPC-Lipo extravasation from CD31^+^ vasculature into GBM regions (**Figure 3G**), validating efficient BBB penetration.

Collectively, our results demonstrate that LPC incorporation endows liposomes with exceptional BBB-penetrating capacity and *in vivo* tumor accumulation, confirming LPC's critical role in enhancing nanocarrier transcytosis. Conventional BBB-penetrating liposomes typically require synthetic phospholipid-ligand conjugates (e.g., DSPE-PEG-Ang/RGD) 15, 33, necessitating complex multi-step synthesis to balance stability, drug-loading, and targeting. While our previous work demonstrated BBB penetration via nicotinic acetylcholine receptor (nAChR) and choline transporter (ChT) using MPC nanogel containing choline and acetylcholine analogues 34, LPC-Lipo retains this targeting capability through endogenous choline head groups of LPC and PC. Crucially, our approach bypasses laborious chemical synthesis by directly integrating LPC into standard liposomal formulations, leveraging its dual role as both a transcytosis activator and structural component. This one-step engineering yields a nanocarrier with superior BBB-penetrating ability. As an endogenous phospholipid, LPC further offers enhanced translational potential over synthetic alternatives.

### LPC-Lipo Triggers p62-mediated Directional Trafficking to the Recycling Pathway for Activated BBB Transcytosis

**Figure 4:**
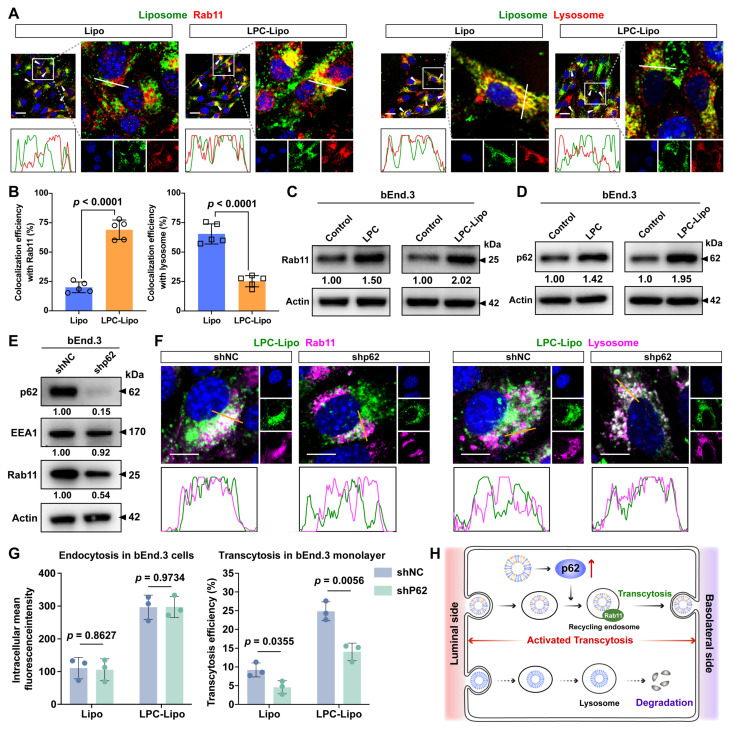
LPC-Lipo activates p62-mediated recycling endosome trafficking to potentiate BBB transcytosis. (A) Confocal images showing co-localization of DiO-labeled Lipo and LPC-Lipo (green) with Rab11^+^ recycling endosome or lysosome (red) in bEnd.3 cells. Intensity profiles along white lines (merged images) demonstrate colocalization peaks. (B) Quantified co-localization efficiency of Lipo and LPC-Lipo with recycling endosome and lysosome (n = 5). (C) WB analysis of Rab11 expression levels in bEnd.3 cells treated with LPC or LPC-Lipo. (D) WB analysis of p62 expression levels in bEnd.3 cells treated with LPC or LPC-Lipo. (E) WB analysis of Rab11 expression levels in p62-KD bEnd.3 cells. (F) Confocal images showing co-localization of DiO-labeled LPC-Lipo (green) with Rab11^+^ recycling endosome or lysosome (red) in WT and p62-KD bEnd.3 cells. Intensity profiles along white lines (merged images) demonstrate colocalization peaks. (G) Endocytosis and transcytosis efficiency of Lipo and LPC-Lipo in WT and p62-KD bEnd.3 cells (n = 3). (H) Schematic illustration of LPC-Lipo upregulating p62 to trigger directional trafficking to the recycling pathway for transcytosis. Scale bar: 20 μm (A); 10 μm (F). Data are presented as mean ± s.d. Statistical significance was analyzed by unpaired two-tailed Student's *t*-test for B and G.

We have demonstrated that LPC-Lipo enhances BBB penetration and activates endocytosis in bECs. As previously established, BBB transcytosis involves nanoparticle endocytosis into early endosomes followed by sorting into two pathways: one directs to recycling endosomes for basolateral exocytosis to complete transcytosis, while the other leads to lysosomal degradation (**Figure S1A**). Thus, transcytosis efficiency depends critically on intracellular trafficking directions. To investigate whether LPC-Lipo's enhanced BBB penetration involves altered transport routing, we assessed its co-localization with key pathway markers in bEnd.3 cells. Rab11^+^ recycling endosomes are known to mediate the transcytosis of macromolecule to the basolateral side 35, 36. Compared to Lipo, LPC-Lipo exhibited significantly higher co-localization with Rab11^+^ recycling endosomes (69.08% ± 8.27% *vs.* 20.02% ± 4.59% for Lipo; **Figure 4A-B**). Conversely, LysoTracker staining revealed markedly reduced lysosomal accumulation of LPC-Lipo (25.30% ± 4.76% *vs.* 65.42% ± 8.55% for Lipo). Pearson correlation coefficients analysis confirmed stronger co-localization of LPC-Lipo with recycling endosomes (0.57 vs. 0.22 for Lipo) and weaker co-localization with lysosomes (0.38 vs. 0.75 for Lipo) (**Figure S6A**). These results indicate that LPC-Lipo not only enhances endocytosis but also redirects early endosomes toward the recycling pathway, minimizing lysosomal trafficking.

We next explored mechanisms underlying this directional switching. Both LPC and LPC-Lipo significantly upregulated Rab11 protein levels in bEnd.3 cells (**Figure 4C**) and increased Rab11^+^ vesicle density (**Figure S6B**), indicating that LPC enhances the recycling endosome formation and LPC-Lipo retains this bioactivity. We further elucidated the potential mechanism driving this phenomenon. Recycling endosomes arise through tubular extensions and vesicle fission from early endosome, a process requiring vesicle segregation and reorganization 37. Critically, recent studies identify p62/SQSTM1, a key regulator of cellular phase separation 38, in regulating membrane trafficking and vesicle organization 39. Building on this insight, we studied p62's role in mediating intracellular trafficking of LPC-Lipo. We first measured p62 levels in LPC- and LPC-Lipo-treated bEnd.3 cells and observed significant upregulation of p62 expression (**Figure 4D**). Immunofluorescence analysis revealed pronounced co-localization of p62 with both EEA1^+^ early endosome and Rab11^+^ recycling endosome (**Figure S6C**). To explore p62's role in regulating early-to-recycling endosome trafficking, we generated p62-knockdown bEnd.3 cells. While p62 depletion did not alter EEA1 levels, it significantly reduced Rab11 expression and Rab11^+^ vesicle density (**Figure 4E** and **Figure S6D**). Critically, p62 knockdown altered the trafficking direction of LPC-Lipo. Fluorescence imaging showed that, compared to control cells where LPC-Lipo mostly localized to Rab11^+^ recycling endosomes, p62-knockdown cells exhibited reduced Rab11^+^ co-localization and increased lysosomal accumulation of LPC-Lipo (**Figure 4F**). Quantitative analysis confirmed that, upon p62 knockdown, the Pearson correlation coefficient between LPC-Lipo and recycling endosome decreased from 0.67 to 0.29. Functional assays confirmed that p62 knockdown did not impair LPC-Lipo internalization but reduced its transcytosis efficiency by ~43% in an *in vitro* BBB model (**Figure 4G**). These findings establish that p62 mediates early-to-recycling endosome trafficking to regulate BBB transcytosis. LPC-Lipo activates transcytosis through p62 upregulation to drive preferential trafficking toward the recycling pathway (**Figure 4H**). Notably, even with p62 knockdown, LPC-Lipo retained higher transcytosis than Lipo, attributable to its endocytosis-enhancing property.

Collectively, LPC-Lipo achieves superior BBB penetration through dual mechanisms: (1) enhanced endocytosis and (2) p62-mediated redirection toward the recycling pathway. While most brain-targeting nanoparticles rely on receptor-mediated BBB recognition, they often suffer lysosomal entrapment in bECs, limiting efficient transcytosis 40. LPC-Lipo overcomes this limitation by coupling endocytosis activation with intracellular trafficking reprogramming. Our study identifies a previously unrecognized regulatory role of p62 in BBB transcytosis. Although the molecular details of p62-mediated vesicle reorganization (e.g., protein complexes assembly) warrant further investigation, this work provides a novel perspective for BBB-penetrating nanoparticle engineering.

### LPC-Lipo Enables BBB-Penetrating PTX Delivery for GBM Chemotherapy and Immunotherapy

**Figure 5:**
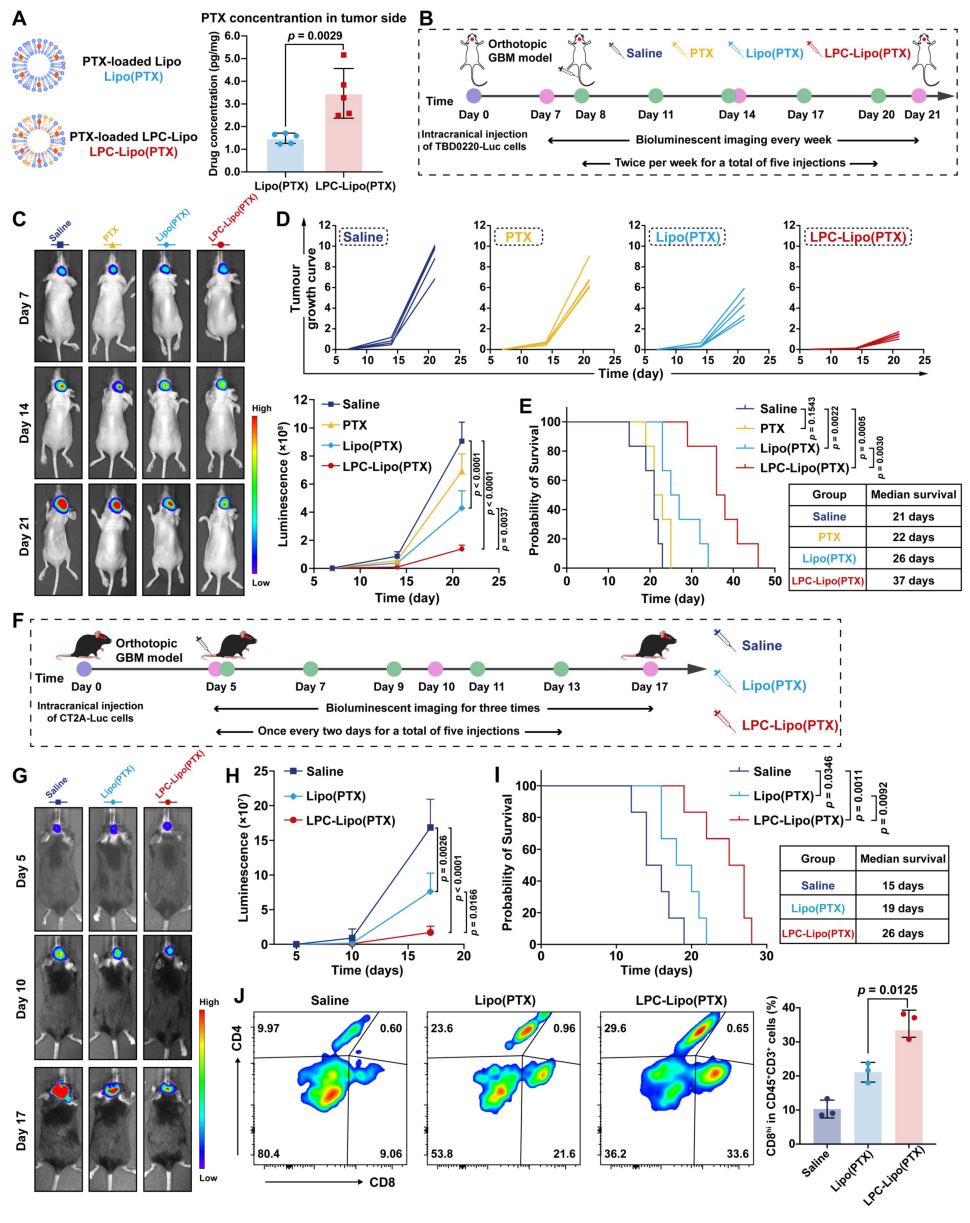
LPC-Lipo facilitates PTX brain delivery for GBM therapy. (A) Schematic of Lipo(PTX) and LPC-Lipo(PTX), with PTX concentrations in GBM tumor side post-intravenous injection (n = 5). (B) Experimental timeline for PDX model establishment and LPC-Lipo(PTX) treatment. (C) Representative *in vivo* bioluminescence images of TBD0220-Luc-bearing mice at days 7, 14, and 21 post-implantations. (D) Individual and summarized tumor growth curves quantified by bioluminescence intensities (n = 5). (E) Kaplan-Meier survival curves of TBD0220-bearing mice with indicated treatments (n = 6). (F) Experimental timeline for orthotopic CT2A GBM model establishment and treatment. (G) Representative *in vivo* bioluminescence images of CT2A tumors at days 5, 10, and 17. (H) CT2A tumor growth curves based on bioluminescent signal quantification (n = 5). (I) Survival analysis of CT2A-bearing mice with indicated treatments (n = 6). (J) Flow cytometry analysis (representative plots and quantification) of tumor-infiltrating CD8^+^ T cell in the indicated groups (n = 3). Data are presented as mean ± s.d. Statistical significance was analyzed by unpaired two-tailed Student's *t*-test for A, two-way ANOVA with Tukey's test for D and H, log-rank test for E and I, and one-way ANOVA with Tukey's multiple comparisons test for J.

Capitalizing on the validated BBB-penetrating capacity of LPC-Lipo, we next engineered a novel PTX-loaded liposomal formulation utilizing this platform, and evaluated its delivery efficiency and therapeutic potential against GBM. PTX was encapsulated into both Lipo and LPC-Lipo, yielding formulations named Lipo(PTX) and LPC-Lipo(PTX) (**Figure 5A**). TEM and DLS characterization confirmed that LPC-Lipo(PTX) displayed a spherical vesicular morphology with a hydrodynamic diameter of 139.5 nm, a PDI of 0.210 ± 0.017, and a zeta potential of -30.44 ± 2.76 mV (**Figure S7A-C**). The formulation exhibited excellent colloidal stability, with no significant changes in size or zeta potential over 7 days at 4°C (**Figure S7D-E**). Furthermore, the DLC (%) and EE (%) of LPC-Lipo(PTX) were 6.62 ± 0.44% and 95.3 ± 3.3%, respectively, comparable to those of Lipo(PTX) (**Figure S7F-G**). This indicates that LPC incorporation did not compromise the drug loading performance of the liposomes. To assess *in vivo* delivery efficiency, we established an orthotopic PDX model. Following intravenous administration, LPC-Lipo(PTX) exhibited significantly higher PTX accumulation in tumors, achieving a 2.3-fold higher PTX concentrations in tumor regions compared to Lipo(PTX) (**Figure 5A**), demonstrating the superior BBB-penetrating and GBM-targeted delivery of LPC-Lipo. The limited PTX accumulation observed with Lipo(PTX) suggests that conventional liposomes rely mainly on passive targeting 41, which exhibit relatively low delivery efficiency. Moreover, we have previously demonstrated elevated expression of choline transporters in GBM tissues compared to normal brain regions 34, which may further enhance the GBM-targeted accumulation of LPC-Lipo(PTX). Collectively, these findings establish LPC-Lipo as an effective strategy for BBB-penetrating PTX delivery to GBM.

We subsequently evaluated the therapeutic efficacy of LPC-Lipo(PTX) in an orthotopic TBD0220-Luc GBM model. Tumor-bearing mice were randomized into four groups receiving intravenous injections of saline, free PTX, Lipo(PTX), and LPC-Lipo(PTX) every three days for five cycles (**Figure 5B**). Tumor progression was monitored by bioluminescence imaging (BLI) (**Figure 5C-D**). Quantitative BLI analysis revealed that free PTX showed negligible tumor suppression, paralleling saline controls and confirming its poor BBB penetration. In contrast, LPC-Lipo(PTX) significantly inhibited tumor progression, reducing bioluminescence intensity to 15.3% the saline controls by day 21. The limited therapeutic improvement observed in the Lipo(PTX) group, which is consistent with its previously demonstrated insufficient BBB penetration and tumor accumulation. Survival analysis revealed LPC-Lipo(PTX) treatment extended median survival to 37 days, representing a 42% increase over the Lipo(PTX) group (**Figure 5E**). H&E-stained brain sections from LPC-Lipo(PTX)-treated mice showed minimal tumor areas with defined margins compared to controls (**Figure S8A**). Taken together, LPC-Lipo(PTX) significantly inhibits GBM progression and prolongs survival.

Beyond its cytotoxic effects, PTX is known to stimulate an immune-reactive tumor microenvironment (TME). Given that GBM is characterized by profound immunosuppression, we evaluated whether LPC-Lipo(PTX) could elicit antitumor immune responses in addition to its therapeutic efficacy in an orthotopic CT2A-Luc GBM model (**Figure 5F**). Tumor burden and survival were monitored by BLI and survival analysis (**Figure 5G-I** and **Figure S8B**). Saline-treated mice exhibited rapid and aggressive tumor progression with a median survival of only 15 days. Notably, LPC-Lipo(PTX) treatment significantly suppressed tumor growth and extended median survival to 26 days. Histological analysis of LPC-Lipo(PTX)-treated brains showed minimal residual tumor and absent cell infiltration (**Figure S8C**). Immunophenotyping revealed 3.2-fold increased CD8^+^ T-cell infiltration in LPC-Lipo(PTX) treated brain tumors compared to saline controls, alongside increased CD4^+^ T-cell populations (**Figure 5J**). These findings align with previous reports that PTX triggers cytotoxic T cell infiltration and activation 42, suggesting that the observed GBM suppression and immune activation are directly attributable to efficient PTX delivery into GBM regions. Collectively, the LPC-Lipo platform effectively enables PTX delivery across the BBB to GBM tissues, establishing LPC-Lipo(PTX) as a novel and potent therapeutic agent for GBM.

Given the importance of nanocarrier biosafety for GBM therapy, we comprehensively evaluated the systemic and neurological toxicity of LPC-Lipo and LPC-Lipo(PTX). Hematological analysis confirmed that key parameters, including red blood cells (RBCs), hemoglobin (HGB), white blood cells (WBCs), granulocyte percentage (Gran%), and platelets (PLTs), remained within normal physiological ranges (**Table S1**), indicating no significant hemotoxicity or inflammatory reactions. Serum biochemistry further showed unaltered levels of hepatic function markers (ALT, AST, ALP, TBIL) and renal function markers (CREA, UREA) (**Table S2**), confirming the absence of hepatorenal toxicity. Histopathological examination (H&E staining) of major organs (heart, liver, spleen, lung, and kidney) revealed no significant pathological changes (**Figure S9**). We further evaluated neurobehavioral functions using two well-established methods. The open field test showed no impairment in motor function, as measured by total distance traveled, or in exploratory behavior, as reflected by time spent in the central area. Additionally, the novel object recognition test indicated no deficits in learning and memory, as assessed by the recognition index (**Figure S10**). Collectively, these results demonstrate that LPC-Lipo exhibits an excellent safety profile with no detectable systemic or neurotoxic effects, supporting its biocompatibility for GBM therapy.

### LPC Functionalization Empowers Lipusu^®^ for GBM Chemotherapy

**Figure 6:**
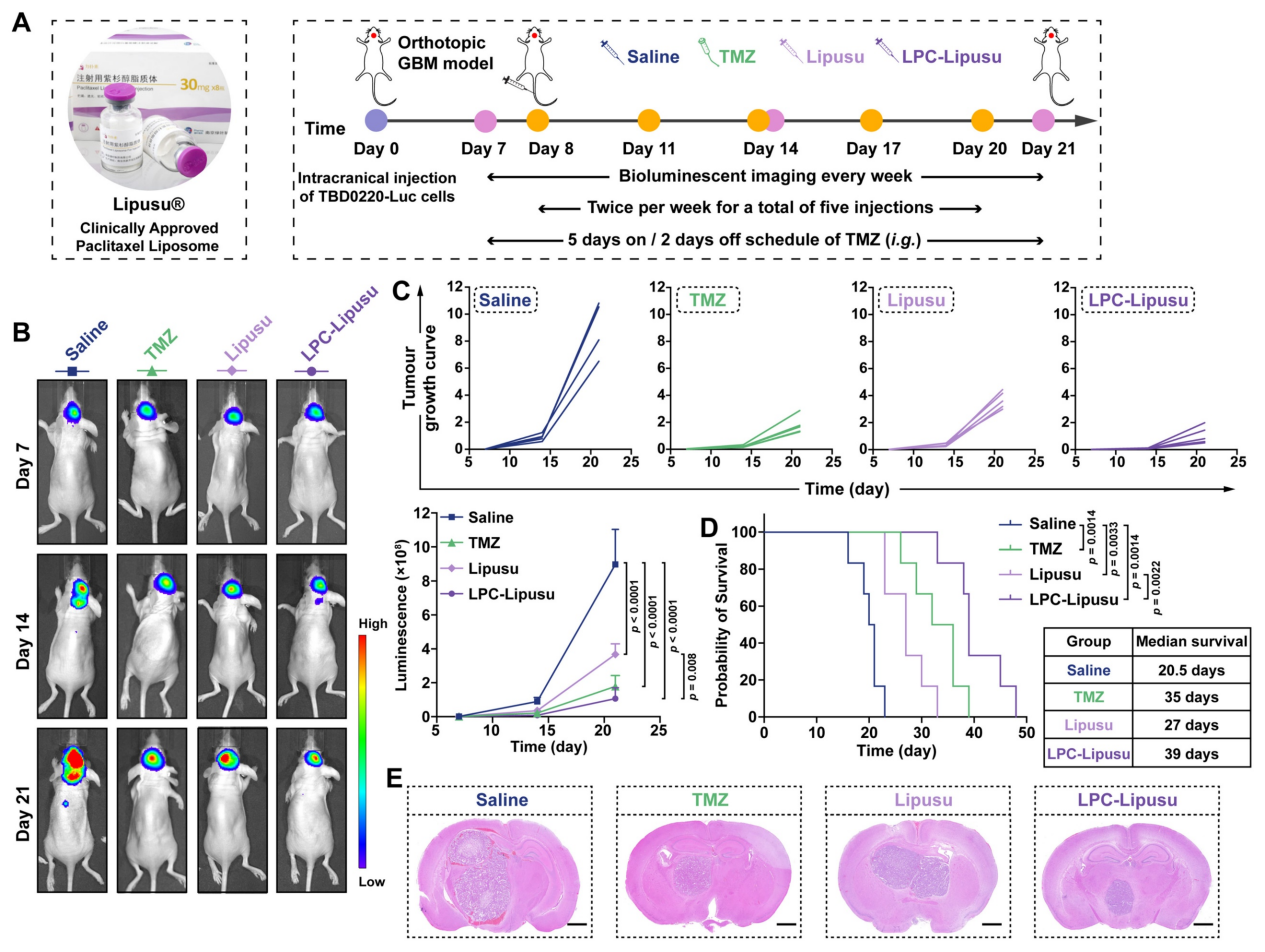
LPC-functionalized Lipusu^®^ potentiates GBM chemotherapy efficacy. (A) Experimental timeline for TBD0220-Luc tumor implantation and LPC-Lipusu treatment in PDX model. (B) Representative *in vivo* bioluminescence images of tumor-bearing mice at days 7, 14, and 21 post-implantations. (C) Individual and summarized tumor growth curves quantified by bioluminescence intensity (n = 5). (D) Kaplan-Meier survival curve of TBD0220-bearing mice with different treatments (n = 6). (E) Representative images of H&E-stained whole-brain sections from PDX model mice with indicated treatments. Scale bar: 100 μm. Data are presented as mean ± s.d. Statistical significance was analyzed by two-way ANOVA with Tukey's test for C and log-rank test for D.

Lipusu^®^, developed by Nanjing Luye Pharmaceutical Co., Ltd., is the first commercially approved PTX liposomal formulation globally. Its established efficacy and safety profile, validated in over 2 million patients, have led to its recommendation as a first-line chemotherapy in the Chinese Society of Clinical Oncology (CSCO) guidelines for lung and breast cancer. Building upon our findings that LPC-Lipo facilitates PTX delivery to GBM for therapeutic benefit, we sought to validate this strategy using the clinically approved Lipusu^®^ platform. LPC-functionalized Lipusu^®^ (LPC-Lipusu) was prepared by incorporating LPC molecules into Lipusu^®^ through hydrophobic interactions between the LPC lipid tail and the liposomal bilayer during a simple incubation step. DLS and TEM results confirmed that LPC-Lipusu maintained a spherical vesicular structure, with a particle size of 166 nm and a zeta potential of -9.2 ± 1.6 mV. It demonstrated excellent colloidal stability over 7 days, with no significant changes in particle size or zeta potential. Furthermore, quantitative PTX content analysis confirmed that LPC insertion did not compromise the PTX loading (**Figure S11**). We then evaluated the antitumor efficacy of LPC-Lipusu in an orthotopic TBD0220-bearing GBM model, using TMZ, the clinical gold standard for GBM therapy, as a control (**Figure 6A**). BLI analysis revealed that while Lipusu^®^ treatment reduced signal intensity compared to the saline control, indicating a therapeutic response to GBM, its efficacy was less pronounced than that of TMZ (**Figure 6B-C**). This limitation is primarily attributable to Lipusu^®^'s restricted BBB penetration. In striking contrast, LPC-Lipusu treatment resulted in minimal tumor burden, reducing bioluminescence intensity to 46.3% of the Lipusu^®^ group by day 21, indicating significantly enhanced GBM proliferation inhibition. Survival analysis further underscored the superiority of LPC-Lipusu: the median survival ranked as LPC-Lipusu (39 days) > TMZ (35 days) > Lipusu^®^ (27 days) > saline (20.5 days) (**Figure 6D**). Histological analysis of H&E-stained whole-brain sections confirmed the efficacy of LPC-Lipusu in reducing tumor size (**Figure 6E**). These results indicate that LPC functionalization effectively transforms Lipusu^®^ into a potent GBM chemotherapy strategy. Considering Lipusu^®^'s well-documented safety and tolerability profile from extensive clinical use, coupled with the inherent biocompatibility of LPC as a natural lipid, this simple one-step functionalization approach not only preserves the favorable safety of Lipusu^®^ but also confers potent therapeutic efficacy against GBM. This strategy thus holds promise for expanding the clinical indications of Lipusu^®^. It is noteworthy, however, that precise modulation of the LPC-to-liposome ratio and process optimization warrant further investigation to ensure scalable manufacturing and clinical-grade reproducibility.

Collectively, results obtained using both self-developed and commercial systems demonstrate that LPC-Lipo serves as effective carriers for BBB-penetrating PTX delivery to GBM. This establishes a foundation for subsequent exploration of PTX-sensitized TTFields-based GBM therapy.

### LPC-Lipo(PTX) Sensitizes GBM to TTFields and TMZ through Mitosis Disruption and DNA Damage Repair Inhibition

**Figure 7:**
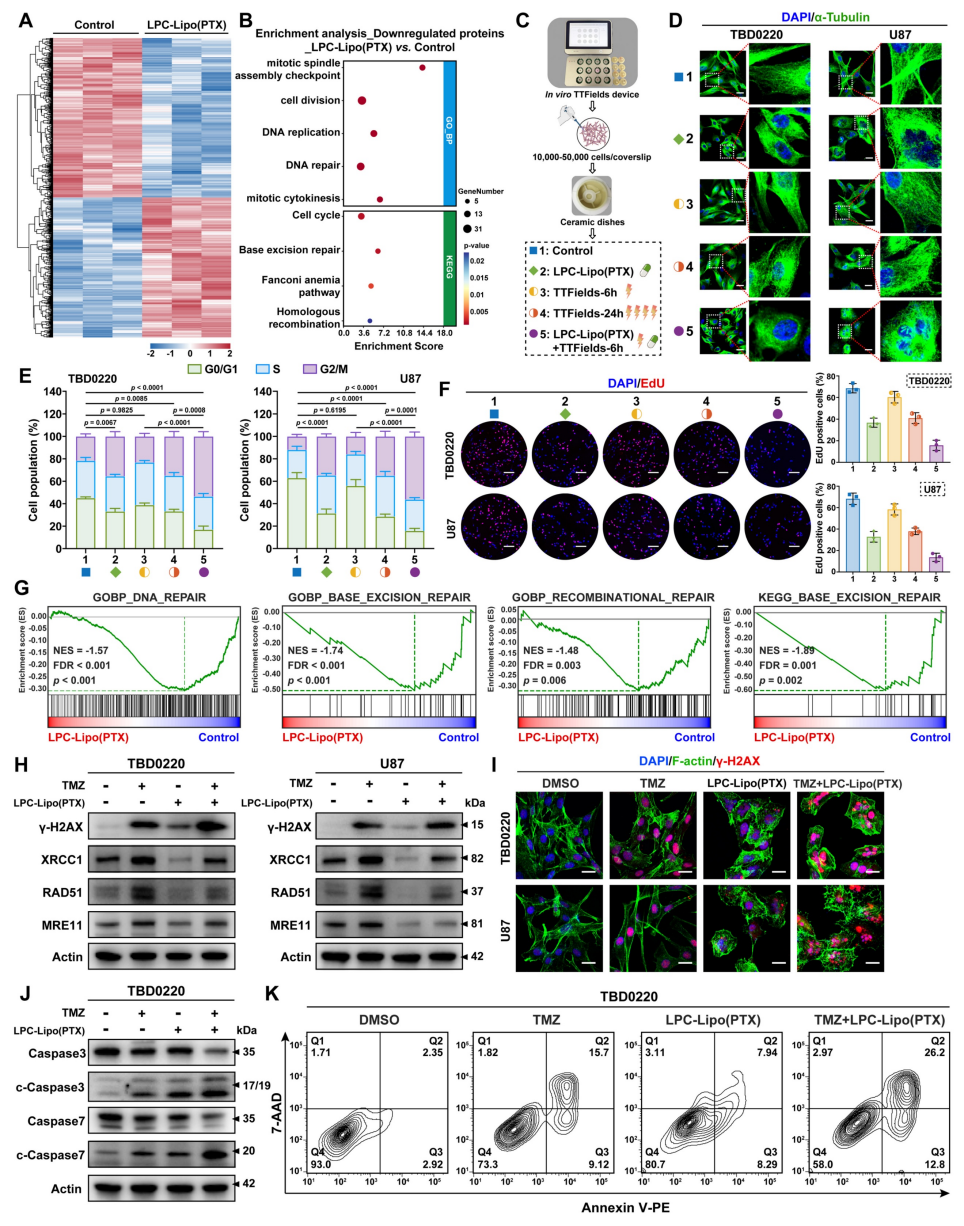
LPC-Lipo(PTX) sensitizes GBM cells to TTFields and TMZ by dual disruption of mitosis and DNA damage repair. (A) Heatmap of differentially expressed proteins in TBD0220 cells post-LPC-Lipo(PTX) treatment versus control. (B) Enrichment analysis of downregulated proteins following LPC-Lipo(PTX) treatment. (C) *In vitro* TTFields device schematic and combination treatment regimen with LPC-Lipo(PTX). (D) Immunofluorescence staining of α-tubulin in TBD0220 and U87 cells with indicated treatment. (E) Cell cycle detection in TBD0220 and U87 cells with indicated treatment (n = 3). (F) EdU staining images and quantification of EdU positive cells ratios in TBD0220 and U87 cells with indicated treatments (n = 3). (G) GSEA analysis of differentially expressed proteins between LPC-Lipo(PTX) and control group using GO-BP and KEGG gene sets related to DNA damage repair. (H) WB analysis of γ-H2AX and DNA damage repair proteins in TBD0220 and U87 cells with indicated treatments. (I) Immunofluorescence images of γ-H2AX in TBD0220 and U87 cells with indicated treatments. (J) WB analysis of Caspase-3/7 and cleaved-Caspase-3/7 in TBD0220 cells with indicated treatments. (K) Apoptosis of TBD0220 cells with indicated treatments analyzed by flow cytometry. Scale bar: 20 μm (D, I); 100 μm (F). Data are presented as mean ± s.d. Statistical significance was analyzed by two-way ANOVA with Tukey's test for E.

PTX inhibits tubulin depolymerization during mitosis, potentially synergizing with TTFields-induced microtubule alignment disruption and mitosis arrest. Building upon the confirmed intratumoral delivery and therapeutic efficacy of LPC-Lipo(PTX) in GBM models, we investigated its potential to sensitize GBM cells to the TTFields/TMZ regimen. Proteomic profiling of LPC-Lipo(PTX)-treated TBD0220 cells (a patient-derived GBM cell line) identified 432 upregulated and 492 downregulated proteins ((|Log_2_(fold change)| > 0.26, *p* < 0.05, FDR-adjusted; **Figure 7A**). Subsequent GO and Kyoto Encyclopedia of Genes and Genomes (KEGG) enrichment analysis revealed that the downregulated proteins were significantly enriched in: (1) biological processes related to cell division, mitotic cytokinesis, and cell cycle, suggesting potential synergy with TTFields; and (2) DNA repair pathways, including base excision repair and homologous recombination, indicative of potential TMZ sensitization (**Figure 7B**). We further studied the sensitization effects of LPC-Lipo(PTX) based on these pathways.

We first evaluated the combined effects of LPC-Lipo(PTX) and TTFields *in vitro* using a system consisting conductive ceramic dishes, a culture base plate, and an electric field generator. Cells seeded on glass coverslips within the dishes were exposed to alternating electric fields along orthogonal axes. Five groups were established, including standard TTFields (24 h) and LPC-Lipo(PTX) combined with shortened TTFields duration (6 h) (**Figure 7C**). Immunofluorescence staining of α-tubulin was performed to assess microtubule morphology and distribution in TBD0220 and U87 cells (**Figure 7D**). Control cells exhibited uniformly aligned microtubules, whereas LPC-Lipo(PTX) induced characteristic microtubule bundling and stabilization. A 6-hour TTFields treatment alone was insufficient to alter microtubule organization, while significant disarray requiring 24 h of exposure. Notably, the combination of LPC-Lipo(PTX) with only 6 h of TTFields induced a more profound microtubule perturbation, characterized by shortened, thickened bundles and a diffuse cytoplasmic signal. Flow cytometric analysis demonstrated that both LPC-Lipo(PTX) and 24-hour TTFields induced G2/M arrest, whereas a 6-hour TTFields exposure alone was ineffective. Notably, the combination of LPC-Lipo(PTX) with only 6 h of TTFields resulted in the most significant G2/M arrest (**Figure 7E**). Consistently, assessment of G2/M-related proteins (CDK1, CyclinB, and its upstream factor p21) revealed maximal p21 elevation and CDK1/Cyclin B suppression in the LPC-Lipo(PTX) plus 6 h TTFields group (**Figure S12A**). EdU proliferation assays further confirmed that the combination of LPC-Lipo(PTX) with only 6 h TTFields significantly inhibited GBM cell proliferation (**Figure 7F**). Collectively, these results demonstrated that LPC-Lipo(PTX) synergizes with TTFields to disrupt mitosis, enabling effective suppression of GBM cell proliferation while reducing the required TTFields exposure duration.

Given the proteomic analysis indicating LPC-Lipo(PTX)-induced downregulation of DNA repair pathways, we performed Gene Set Enrichment Analysis (GSEA) on the differentially expressed proteins. Results confirmed significant downregulation of DNA damage repair (DDR), particularly base excision repair (BER) and homologous recombination (HR) repair (**Figure 7G**), suggesting that LPC-Lipo(PTX) may enhance TMZ efficacy by inhibiting DDR. To validate this, TBD0220 and U87 cells were treated with DMSO, TMZ, LPC-Lipo(PTX), or TMZ+LPC-Lipo(PTX). Western blot analysis showed that while TMZ treatment increased levels of the DNA damage marker γ-H2AX, it concurrently upregulated key DDR proteins (XRCC1, RAD51, MRE11) in both GBM cells (**Figure 7H**). Critically, LPC-Lipo(PTX) treatment downregulated these DDR factors. Moreover, the TMZ+LPC-Lipo(PTX) combination attenuated the TMZ-induced upregulation of DDR proteins while further elevating γ-H2AX levels beyond TMZ alone, indicating suppression of TMZ-triggered DNA repair responses and consequently enhanced DNA damage. Confocal immunofluorescence analysis corroborated these findings (**Figure 7I**). Consistent with increased DNA damage, apoptosis assays revealed higher expression levels of cleaved-Caspase3/7 in TMZ+LPC-Lipo(PTX) treated cells compared to TMZ alone (**Figure 7J** and **Figure S12B**). Flow cytometry quantification confirmed significantly increased apoptotic rates: TMZ induced 24.7% apoptosis in TBD0220 cells, while TMZ+LPC-Lipo(PTX) increased apoptosis to 39%. (**Figure 7K**). A similar enhancement was observed in U87 cells (25.3% with TMZ *vs.* 46% with TMZ+LPC-Lipo(PTX)) (**Figure S12C**). These data demonstrate that LPC-Lipo(PTX) enhances the TMZ sensitivity of GBM cells by inhibiting DNA damage repair, leading to exacerbated DNA damage and apoptosis.

TTFields+TMZ regimen represents the major clinical breakthrough in GBM therapy. Leveraging PTX's microtubule-disrupting properties, we provide a novel strategy to enhance TTFields efficacy by potentiating mitotic arrest. Moreover, although PTX/TMZ co-delivery has been explored, their underlying mechanisms remain incompletely characterized. Recent studies reported PTX augmenting TMZ sensitivity by disrupting XPC and ERCC1 expression and localization 43. Here, we provide evidence that LPC-Lipo(PTX) exerts an inhibitory effect on DNA damage repair, thereby enhancing TMZ-induced DNA damage. These dual sensitization mechanisms support LPC-Lipo(PTX) as a promising adjunct to the TTFields+TMZ regimen, potentially augmenting therapeutic efficacy while reducing TTFields exposure requirements.

### TALECT Regimen Achieves Potent GBM Suppression with Reduced TTFields Exposure *In Vivo*

**Figure 8:**
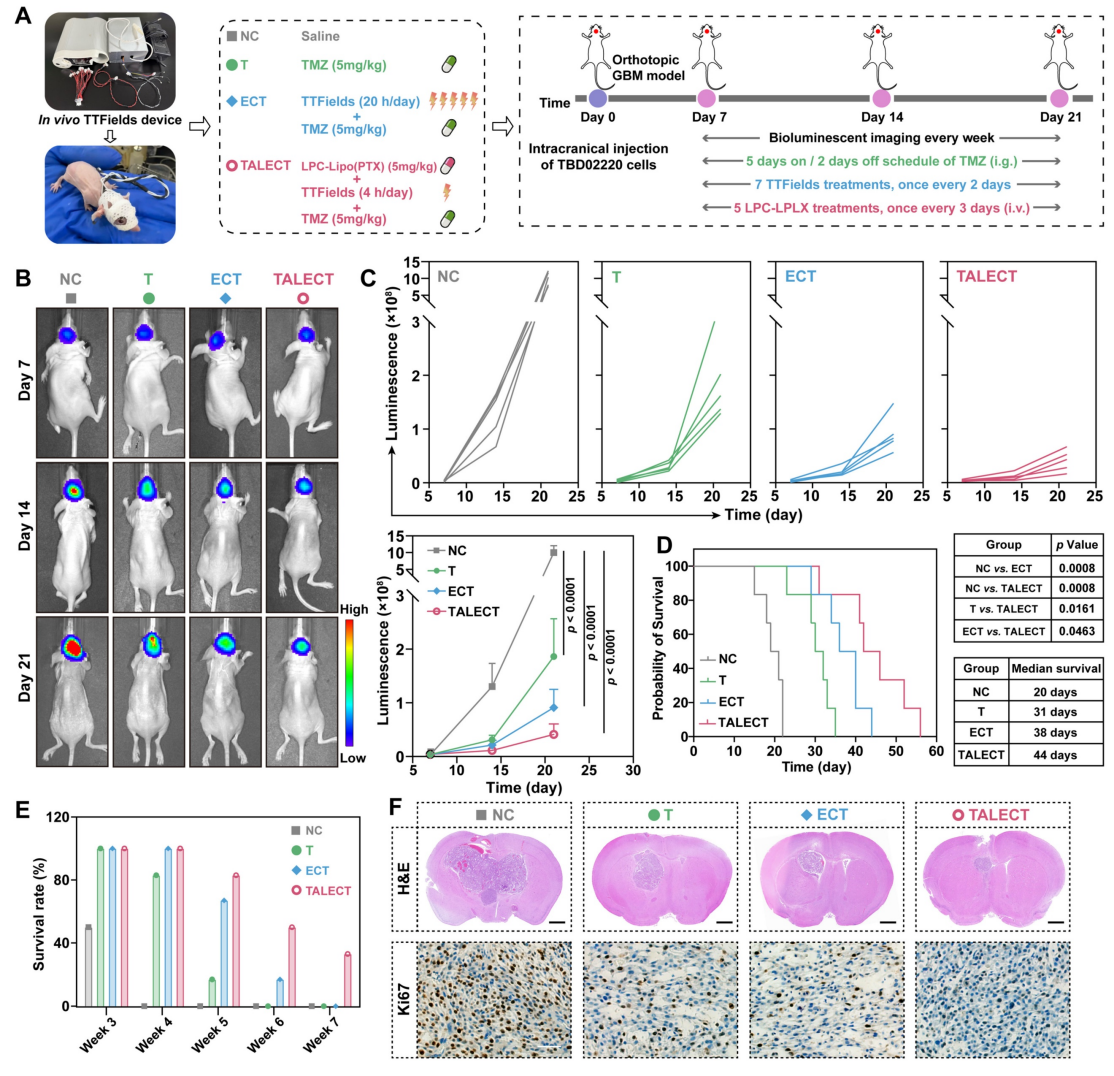
TALECT regimen enables effective GBM therapy with shortened TTFields exposure duration. (A) *In vivo* TTFields device schematic and experimental timeline for TBD0220-Luc tumor implantation and combination treatment. (B) Representative *in vivo* bioluminescence images of tumor-bearing mice at days 7, 14, and 21 post-implantations. (C) Individual and summarized tumor growth curves quantified by bioluminescence intensity (n = 5). (D) Kaplan-Meier survival curve of TBD0220-bearing mice with different treatments (n = 6). (E) Weekly survival probability of mice with different treatment. (F) Representative images of H&E and Ki67-stained brain sections from PDX model mice post-treatments. Scale bar: 100 μm. Data are presented as mean ± s.d. Statistical significance was analyzed by two-way ANOVA with Tukey's test for C and log-rank test for D.

Building upon our development of LPC-Lipo(PTX) and its demonstrated ability to synergistically enhance TTFields and TMZ efficacy *in vitro*, we next performed definitive *in vivo* studies to test the hypothesis that combining LPC-Lipo(PTX) with TTFields/TMZ achieves effective GBM therapy while substantially reducing TTFields exposure duration. Orthotopic GBM models were established using TBD0220-Luc cells. The *in vivo* TTFields system comprised transducer arrays, conductive hydrogel, wiring, and an electric field generator, with four arrays secured in a cross-configuration on the murine cranium for TTFields administration (**Figure 8A**). Treatment groups were as follows: (1) NC: Saline, (2) T: TMZ, (3) ECT ("EC" denotes electric fields): TTFields (20 h/day) + TMZ, and (4) TALECT ("TAL" denotes paclitaxel liposomes): LPC-Lipo(PTX) + TTFields (4 h/day) + TMZ.

Tumor progression was monitored via bioluminescence imaging (**Figure 8B-C**). Compared to TMZ monotherapy (T group), the addition of standard-duration TTFields (20 h/day; ECT group) enhanced GBM growth inhibition. Crucially, the TALECT regimen, incorporating LPC-Lipo(PTX) with only 4 h/day TTFields, achieved superior growth suppression, evidencing lower bioluminescence intensity than the ECT group by day 21. This demonstrates that the triple combination therapy effectively inhibits GBM progression with a 75% reduction in TTFields exposure duration. Given that median survival time and survival rate are primary clinical endpoints for GBM therapy, we conducted survival analysis (**Figure 8D-E**). Median survival times were: NC (20 days), T (31 days), ECT (38 days), and TALECT (44 days). Weekly survival rates further highlighted the advantage of TALECT therapy: at week 5, survival was 67% (ECT) *vs.* 83% (TALECT); by week 6, rates declined to 17% (ECT) and 50% (TALECT), with TALECT maintaining 33% survival at week 7. Histopathological analysis of post-treatment brain tissue corroborated these findings. H&E and immunohistochemical (IHC) staining revealed minimal tumor size and significantly reduced Ki67 proliferation index in the TALECT group (**Figure 8F**). Collectively, the TALECT therapy achieved a 75% reduction in the required TTFields exposure duration while providing enhanced suppression of GBM growth and extension of survival.

Although TTFields confer significant clinical benefits in GBM, their efficacy is strongly time-dependent, requiring daily wear exceeding 18 h for at least 28 days. This prolonged exposure poses substantial challenges to patient compliance, increases usage costs, and elevates nursing burdens, thereby limiting patient accessibility. The TALECT strategy presented here reduces the daily TTFields duration to just 4 h while maintaining therapeutic efficacy, offering a novel approach to optimize the clinical feasibility and cost-effectiveness of TTFields-based regimens.

## Conclusions

In conclusion, we present TALECT therapy, an integrated regimen combining a novel transcytosis-activating PTX liposomes with a shortened TTFields schedule, as a significant advance for GBM. The principal innovation lies in the development of LPC liposomes. Existing BBB penetrating nanocarriers, relying on ligand-receptor interaction-mediated active transport, are hindered by the low transcytosis activity of bECs. LPC-Lipo addresses this bottleneck by activating endothelial transcytosis via two mechanisms: (1) LPC-enhanced membrane plasticity facilitating accelerated endocytosis; and (2) p62-mediated directional trafficking toward Rab11^+^ recycling endosomes, evading lysosomal degradation. This streamlined modification of conventional liposomes establishes a potent BBB-penetrating platform, enabling superior intratumoral PTX delivery and GBM therapy efficacy. Critically, this strategy demonstrates clinical translatability through successful functionalization of Lipusu^®^, which acquires potent anti-GBM activity via LPC incorporation.

Building upon this enhanced delivery, TALECT therapy combines LPC-Lipo(PTX) with the clinical TTFields/TMZ regimen. Leveraging PTX's dual sensitization mechanisms, mitotic arrest amplifying TTFields efficacy and DNA damage repair inhibition potentiating TMZ, TALECT therapy achieves superior GBM growth inhibition and survival benefits while reducing daily TTFields exposure by 75% (from 20 h to 4 h). This reduction directly addresses patient compliance, cost, and accessibility barriers associated with prolonged TTFields.

This study advances the design of BBB-penetrating nanocarriers, offering an innovative platform for central nervous system-targeted drug delivery. TALECT therapy represents a practical regimen that achieves therapeutic efficacy while drastically minimizing TTFields duration, improving the cost-effectiveness of TTFields-based GBM therapy. Future work will focus on (1) optimizing LPC composition for maximum BBB penetration efficiency, (2) advancing scalable production, and (3) conducting long-term safety assessments to further bolster the clinical translation of TALECT therapy.

## Supplementary Material

Supplementary figures, tables and methods.

## Abbreviations

GBM: glioblastoma
LPC: lysophosphatidylcholine
TTFields: tumor treating fields
TMZ: temozolomide
BBB: blood-brain barrier
PTX: paclitaxel
bECs: brain endothelial cells
PC: phosphatidylcholine
CLSM: confocal laser scanning microscope
FC: flow cytometry
SICM: scanning ion conductance microscopy
FRAP: fluorescence recovery after photobleaching
DLS: dynamic light scattering
PDI: polydispersity index
HPLC: high-performance liquid chromatography
DLC: drug-loading capacity
EE: encapsulation efficiency
TEER: trans-endothelial electrical resistance
FITC: fluorescein isothiocyanate
PDX: patient-derived xenograft
HE: hematoxylin and eosin
TEM: transmission electron microscopy
MD: molecular dynamics
WB: western blot
GO: gene ontology
GSEA: gene set enrichment analysis
