# Supplementary material for: LPC-engineered liposomes activate transcytosis for paclitaxel brain delivery and potentiation of TTFields/TMZ via TALECT regimen in glioblastoma
Source: Theranostics. 2026 Jan 1;16(5):2249–68. doi: 10.7150/thno.122658 (PMC12712795; doi:10.7150/thno.122658)
Supplement: Supplementary file 1 — Supplementary figures, tables and methods. [file thnov16p2249s1.pdf]

Supplementary Material

Supplementary Figures

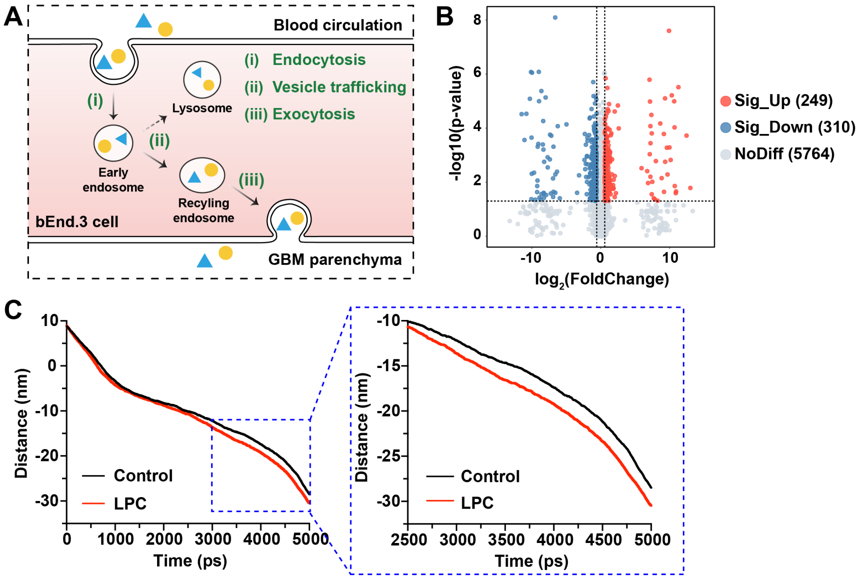

Figure S1. (A) Schematic illustration of the BBB transcytosis process for cargo transport from blood circulation to brain parenchyma, comprising three steps: endocytosis, intracellular trafficking, and exocytosis. (B) Volcano plot of differentially expressed proteins following LPC treatment. Significantly altered proteins (p < 0.05) are labeled (red: upregulated; blue: downregulated). (C) Time-dependent changes in the distance from the protein particle to the initial membrane plane during MD simulations (corresponding to Figure 1F).

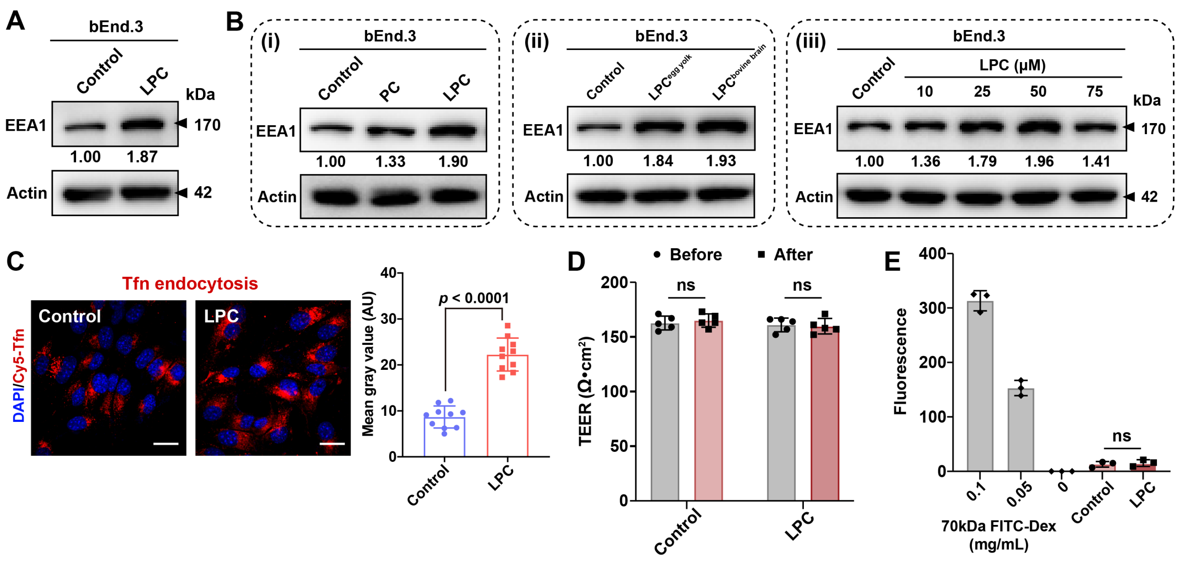

Figure S2. (A) WB analysis of EEA1 expression in LPC-treated bEnd.3 cells. (B) Comparative analysis of EEA1 levels induced by: (i) PC vs. LPC, (ii) LPCs from different sources, and (iii) LPCs at varying concentrations. (C) Confocal images and corresponding quantification of Cy5-Tfn endocytosis in LPC-treated bEnd.3 cells (n = 10). Scale bar: 20 μm. (D) TEER measurements of bEnd.3 monolayer with or without LPC treatment before and after Tfn transcytosis (n = 5). (E) Quantification of 70 kDa FITC-dextran flux across cell-free inserts (first three bars) and bEnd.3 monolayer (control or LPC-treated; last two bars) (n = 3). Data are presented as mean ± s.d.; ns, not significant.

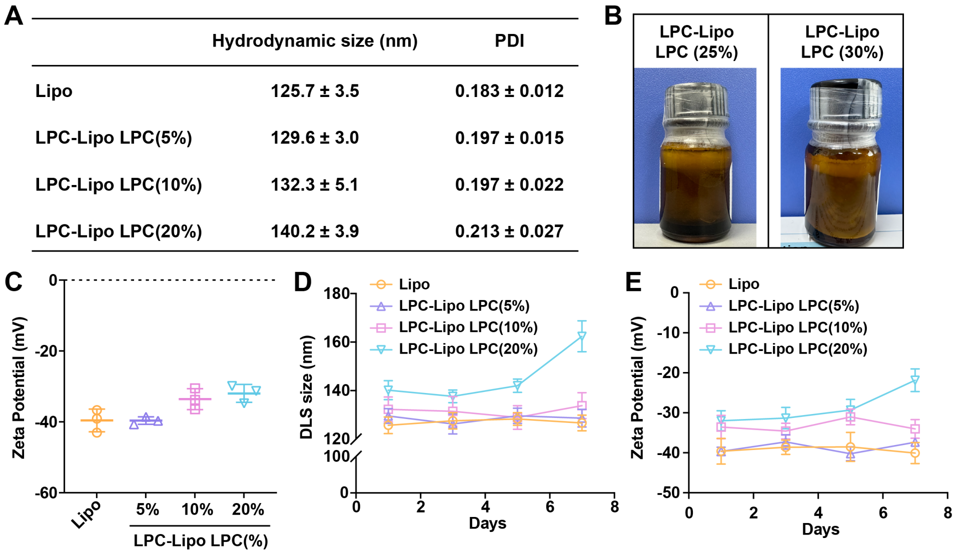

	Hydrodynamic size (nm)	PDI
Lipo	125.7 ± 3.5	0.183 ± 0.012
LPC-Lipo LPC(5%)	129.6 ± 3.0	0.197 ± 0.015
LPC-Lipo LPC(10%)	132.3 ± 5.1	0.197 ± 0.022
LPC-Lipo LPC(20%)	140.2 ± 3.9	0.213 ± 0.027

Figure S3. (A) Hydrodynamic size and PDI of Lipo and LPC-Lipo with varying LPC mass ratios (5%, 10%, and 20%). (B) Visual appearance of LPC-Lipo formulations with 25% and 30% LPC, showing phase separation. (C) Zeta potential of Lipo and LPC-Lipo formulations. (D, E) Colloidal stability of liposomal formulations, assessed by monitoring changes in hydrodynamic size (D) and zeta potential (E) over 7 days. Data are presented as mean ± s.d. (n = 3).

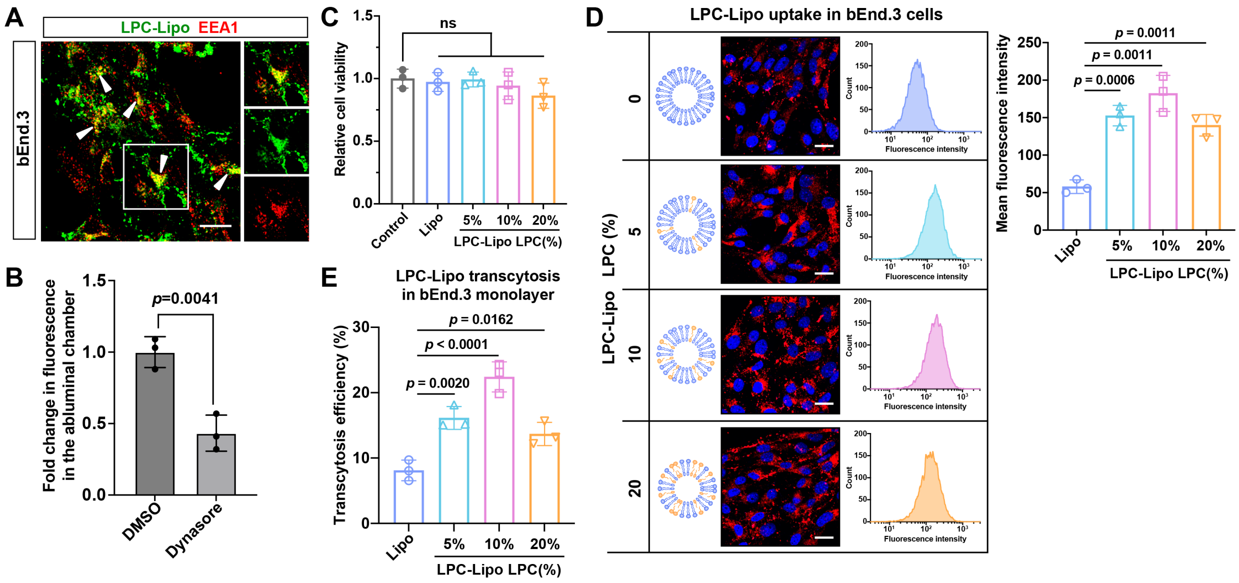

Figure S4. (A) Confocal image showing colocalization of LPC-Lipo (green) with EEA1^{+} early endosome (red). (B) Fold change in fluorescence of LPC-Lipo in the lower chamber of an in vitro BBB model with Dynasore treatment. (C) Cytotoxicity assessment of Lipo and LPC-Lipo in bEnd.3 cells after 24-hour incubation. (D) Cellular uptake efficiency of Lipo and LPC-Lipo with different LPC ratios in bEnd.3 cells. (E) Transcytosis efficiency of Lipo and LPC-Lipo in an in vitro BBB model. Data are presented as mean ± s.d. (n = 3). Scale bar: 20 μm (A, D).

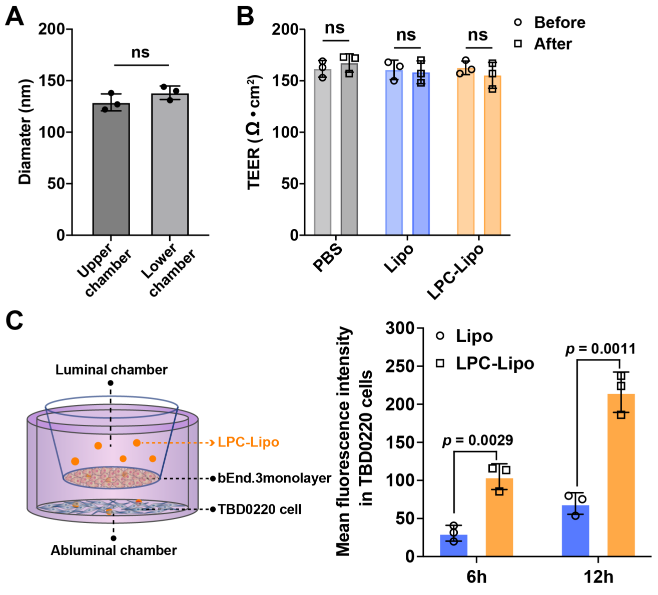

Figure S5. (A) Size distribution of LPC-Lipo in upper and lower chamber of in vitro BBB model. (B) TEER measurements of bEnd.3 monolayer pre- and post-incubation with Lipo and LPC-Lipo. (C) Schematic illustration of the in vitro BBB model with TBD0220 cells seeded in the lower chamber. Flow cytometry quantification of LPC-Lipo entering into TBD0220 cells. Data are presented as mean ± s.d. (n = 3); ns, not significant.

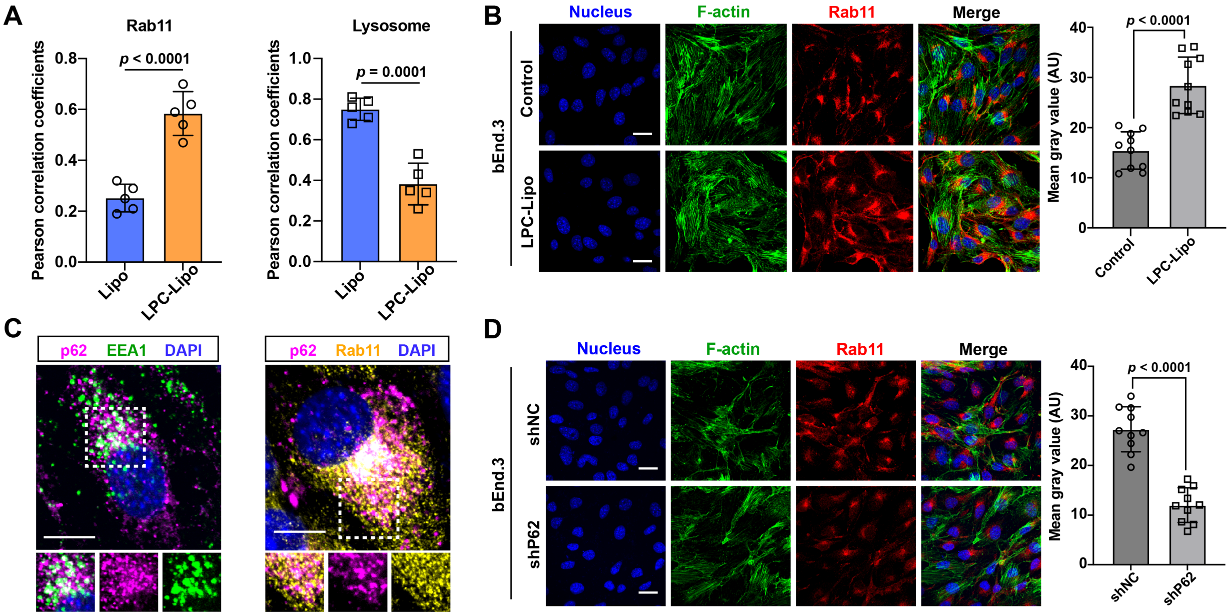

Figure S6. (A) Pearson correlation coefficients between liposome and recycling endosome/lysosome (n = 5). (B) Confocal images and corresponding quantification showing the Rab11^{+} recycling endosome density in bEnd.3 cells post-LPC treatment (n = 10). (C) Confocal images showing co-localization of p62 (pink) with EEA1^{+} early endosome (green) and Rab 11^{+} recycling endosome (yellow) in bEnd.3 cells. (D) Confocal images and corresponding quantification showing the Rab11^{+} recycling endosome density in p62-KD bEnd.3 cells (n = 10). Data are presented as mean ± s.d.

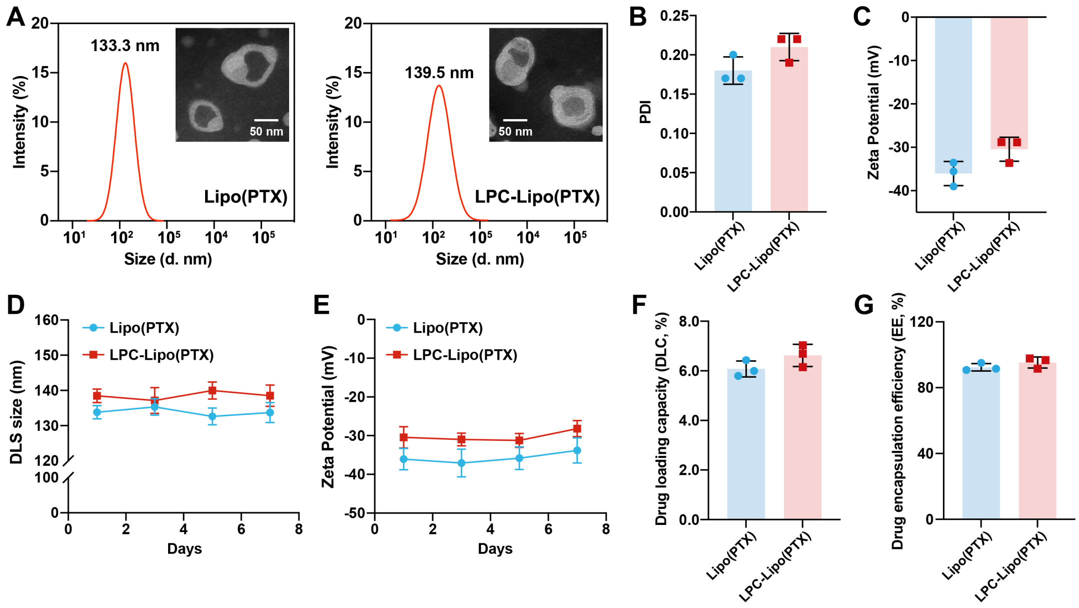

Figure S7. (A) Representative TEM images (scale bar, 50 nm) and corresponding hydrodynamic size distributions of Lipo(PTX) and LPC-Lipo(PTX). (B, C) The PDI (B) and zeta potential (C) of all Lipo(PTX) and LPC-Lipo(PTX), as measured by DLS. (D, E) Colloidal stability profiles monitored through changes in hydrodynamic size (D) and zeta potential (E) over 7 days. (F, G) The drug-loading capacity (F) and drug encapsulation efficiency (G) of Lipo(PTX) and LPC-Lipo(PTX). Data are presented as mean ± s.d. (n = 3).

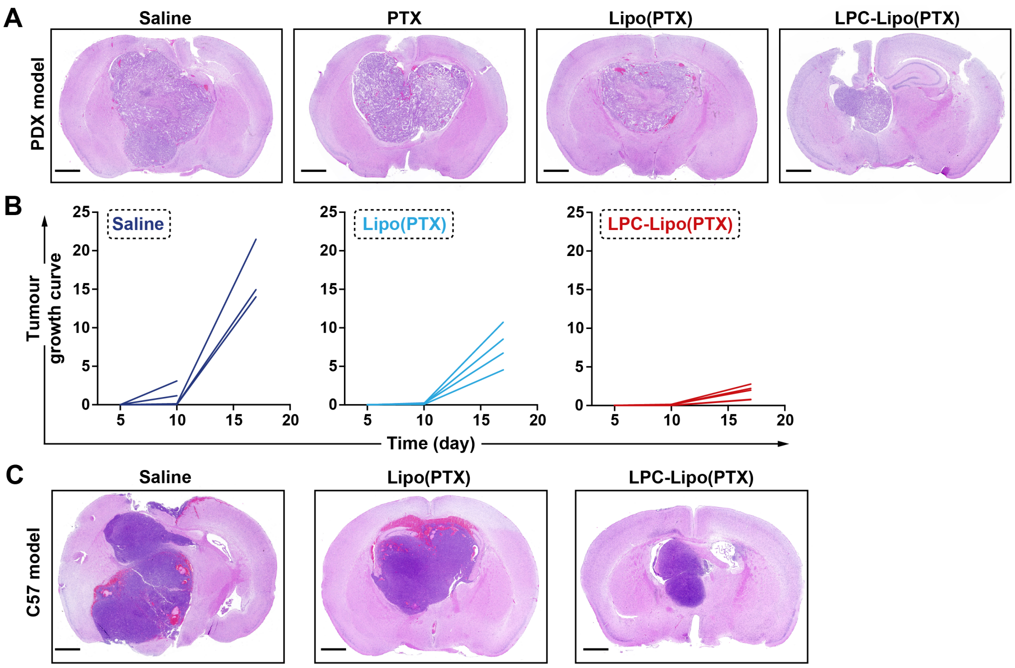

Figure S8. (A) Representative images of H&E-stained whole-brain sections from PDX model mice with indicated treatments. (B) Individual CT2A tumor growth curves quantified by bioluminescence intensity (n = 5). (C) Representative images of H&E-stained whole-brain sections from CT2A-bearing mice with indicated treatments. Scale bar: 100 µm (A, C).

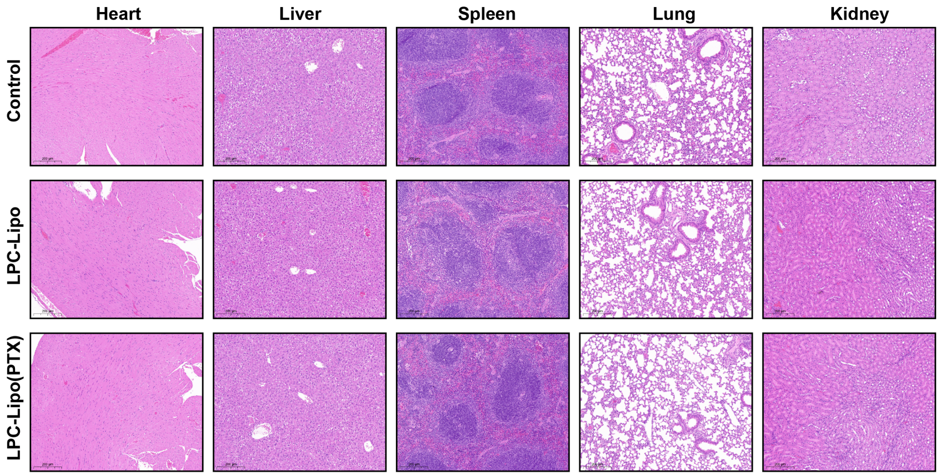

Figure S9. H&E staining of major organs of mice treated with LPC-Lipo and LPC-Lipo(PTX).

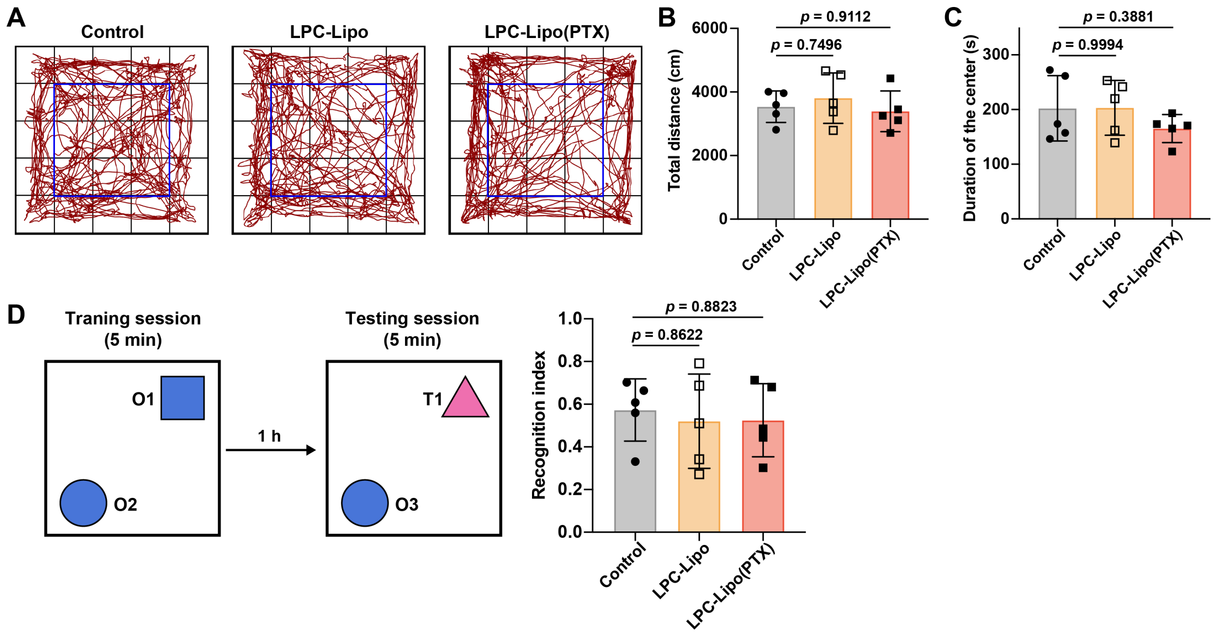

Figure S10. Neurobehavioral assessment of mice treated with LPC-Lipo and LPC-Lipo(PTX). (A) Representative movement trajectories of mice in the open-field test. (B) Total distance traveled in the open-field test. (C) Duration of the center in the open-field test. (D) Schematic of the novel object recognition test procedure. (E) Recognition index calculated in the novel object recognition test. Data are presented as mean ± s.d. (n = 5).

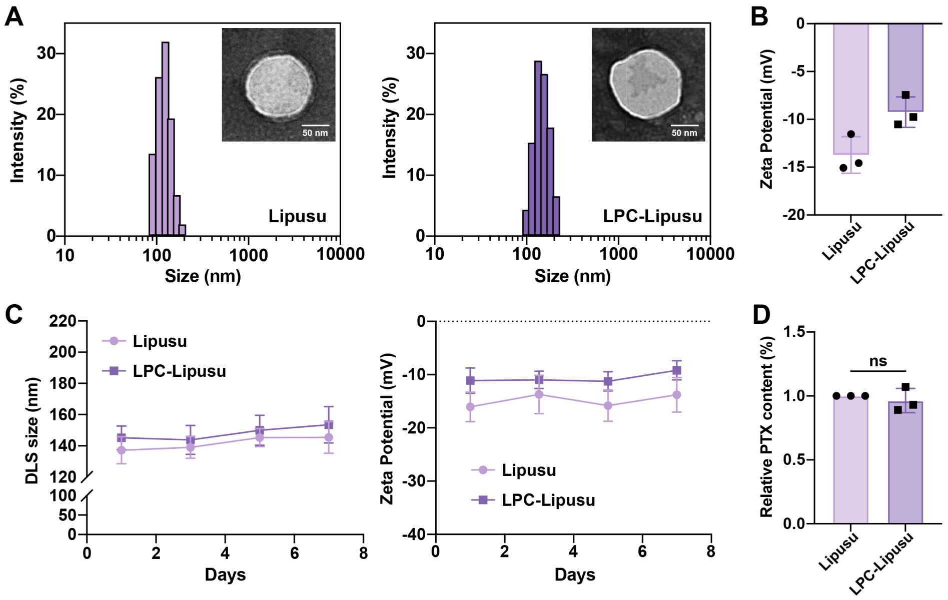

Figure S11. (A) Representative TEM images (scale bar, 50 nm) and hydrodynamic size of Lipusu® and LPC-Lipusu. (B) Zeta potential of Lipusu® and LPC-Lipusu. (C) Colloidal stability profiles monitored through changes in hydrodynamic size and zeta potential over 7 days. (D) Relative PTX content of Lipusu® and LPC-Lipusu. Data are presented as mean ± s.d. (n = 3).

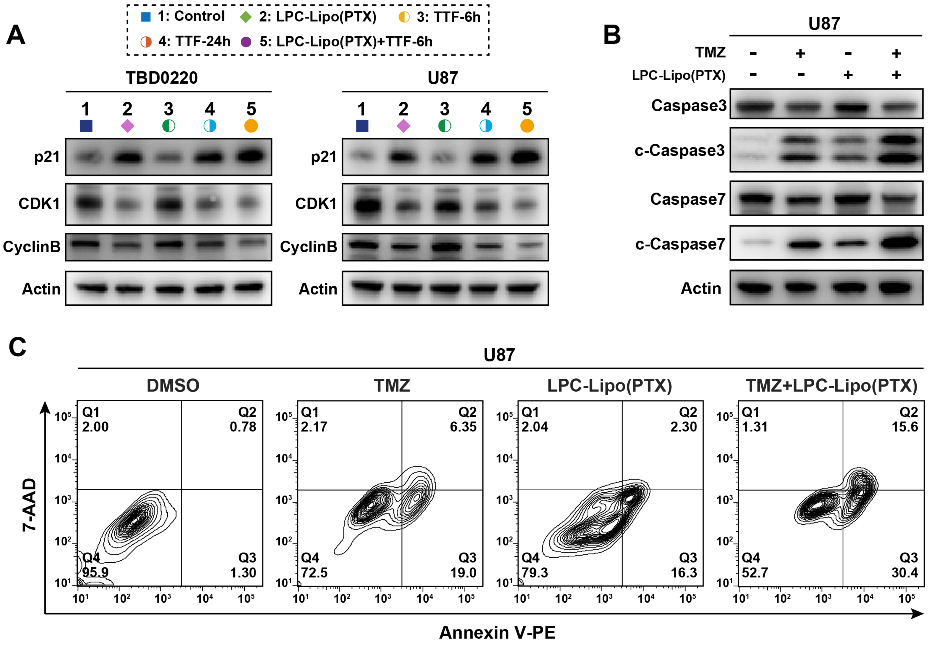

Figure S12. (A) WB analysis of G2/M cycle regulators (p21 and its downstream targets CDK1/Cyclin B) in TBD0220 and U87 cells with indicated treatments. (B) WB analysis of Caspase-3/7 and cleaved-Caspase-3/7 in U87 cells with indicated treatments. (C) Apoptosis of U87 cells with indicated treatments analyzed by flow cytometry.

Supplementary Tables

Table S1. Blood routine analysis in mice treated with LPC-Lipo or LPC-Lipo(PTX).

Group	WBC (10^9/L)	RBC (10^{12}/L)	PLT (10^9/L)	HGB (g/L)	Gran% (%)
**Control**	7.72 ± 0.17	9.85 ± 0.30	851.0 ± 75.1	134.3 ± 2.5	14.97 ± 0.40
**LPC-Lipo**	7.41 ± 0.70	9.93± 0.26	880.4 ± 59.5	132.0 ± 2.6	15.30 ± 1.53
**LPC-Lipo(PTX)**	8.05 ± 0.44	9.68 ± 0.21	928.3 ± 34.4	129.5 ± 3.0	15.18 ± 0.96

Table S2. Serum biochemistry in mice treated with LPC-Lipo or LPC-Lipo(PTX).

Group	ALT (U/L)	AST (U/L)	ALP (U/L)	TBIL (μmol/L)	CREA (μmol/L)	UREA (mmol/L)
**Control**	48.7 ± 8.5	171.6 ± 20.1	140.5 ± 25.6	3.02 ± 0.30	27.5 ± 3.1	9.01 ± 0.06
**LPC-Lipo**	52.4 ± 8.6	190.9 ± 42.1	161.9 ± 22.2	3.14 ± 0.47	25.2 ± 4.9	9.64 ± 1.17
**LPC-Lipo(PTX)**	45.8 ± 4.2	198.2 ± 56.5	132.9 ± 13.7	3.40 ± 0.44	24.3 ± 4.1	8.79 ± 0.37

Supplementary Methods

1. Transmission Electron Microscopy (TEM)

For TEM, cells were scraped, washed twice with PBS, centrifuged, and resuspended in 200 µL plasma for re-centrifugation. After plasma removal, the pellet was fixed with pre-cooled 2.5% glutaraldehyde, treated with 1% osmium tetroxide, dehydrated through a graded ethanol series, and embedded in epoxy resin. Ultrathin sections (70 nm) were stained with 2% uranyl acetate followed by lead citrate and imaged with a HITACHI H-7650 microscope.

2. Molecular Dynamics (MD) Simulation

All MD simulations were performed using the CHARMM-GUI webserver,[1] the Martini coarse-grained force field,[2] and GROMACS software (version 2019.6) to characterize interactions between the protein particle and the cell membrane. The simulated system employed the BSA as a protein model and a planar lipid bilayer (~ 60 nm × 60 nm) modeling cell membrane. The outer membrane leaflet of the bilayer contained PC lipids, while the inner leaflet consisted of phosphatidylethanolamine (PE) and phosphatidylserine (PS). To evaluate the effects of LPC, membrane lipids were partially replaced with LPC in designated systems. Under constant-force conditions, we simulated BSA translocation across the membrane with/without LPC doping. All simulations applied 1.1 nm cutoffs for van der Waals and short-range electrostatic interactions, updated neighbor lists every 20 integration steps, enforced periodic boundary conditions in three dimensions, and used a 20-fs time step under NPT ensemble conditions. Trajectory visualization and rendering were conducted with VMD.[3]

3. Liquid Chromatography-Tandem Mass spectrometry (LC-MS/MS) Proteomics Analysis

Following treatments, cells were lysed with buffer (1% protease inhibitor cocktail), vortexed, sonicated for 5 min, and centrifuged to collect the supernatant as protein extract. Protein

concentration was quantified by BCA assay. Subsequent processing included alkylation, enzymatic digestion, and desalting. Tryptic peptides were separated *via* nanoflow liquid chromatography (Ultimate 3000 RSLC nano system, Thermo Fisher Scientific) and analyzed by high-resolution mass spectrometry using a Q Exactive Plus platform (Thermo Fisher Scientific). Data-independent acquisition (DIA) was performed against the UniProt proteome database without spectral library pre-generation. MS2-level quantification was executed in Spectronaut 17.0 with standard parameters, applying a 1% false discovery rate (FDR) filter at peptide-spectrum match, peptide, and protein group levels. Protein abundance values were $\log_2$-transformed prior to comparative analysis.

4. Cellular Endocytosis Assay

The effect of LPC on endocytosis activity of bEnd.3 cells was studied by using transferrin (Tfn) as a model. Tfn was labeled with Cy5, and cells were pretreated with/without 50 μM LPC for 30 min prior to 4-h Cy5-Tfn incubation. Post-incubation samples underwent triple PBS washing. For confocal imaging, cells were fixed with 4% paraformaldehyde (PFA) and nuclei-stained with DAPI. Images were acquired using a CLSM (FV1200 Olympus, Japan) and quantified with ImageJ. For flow cytometry, cells were trypsinized and resuspended in PBS for analysis.

Liposome endocytosis was systematically analyzed through two complementary approaches: (1) LPC ratio-dependent studies where DiI-labeled LPC-Lipo with varying LPC content (5%, 10%, and 20% w/w of total lipids) were incubated with bEnd.3 cells at 50 μg/mL for 4 h; and (2) time-course analyses in which Lipo and LPC-Lipo designated as optimal (10% LPC) were incubated for 1, 2, 4, and 6 h. Following incubation, all samples underwent identical processing as described for the Tfn assay for both CLSM and flow cytometry analysis. Parallel experiments were

conducted in bEnd.3 cells transfected with either empty vector or p62-knockdown lentivirus (shp62) using identical LPC-Lipo treatment conditions.

5. In Vivo BBB Penetration Imaging

At 14 days post-implantation, the PDX model mice received intravenous injections of DiR-labeled Lipo or LPC-Lipo (1 mg/mL, 100 μL). Brain accumulation was monitored at 2, 4, 6, 8, and 24 h post-injection using an In Vivo Imaging System (IVIS Spectrum, PerkinElmer) with 745 nm excitation / 800 nm emission. For *ex vivo* analysis, mice were sacrificed at 12 h with or without transcardial PBS perfusion. Excised brains were immediately imaged by IVIS, and fluorescence intensity was quantified using Living Image® 4.5 software (Caliper Life Sciences).

For immunofluorescence (IF) analysis, fresh brain tissues were fixed with 4% PFA, embedded in optimal cutting temperature compound (OCT, Sakura Finetek), and cryosectioned at 7 μm thickness. Sections were permeabilized in 0.1% Triton X-100, blocked with 10% FBS, and incubated overnight at 4°C with anti-CD31 antibody (Abcam, #ab28364). After washing, sections were incubated with Alexa Fluor 488-conjugated secondary antibody (Invitrogen) for 1 h at room temperature. Nuclei were counterstained with DAPI (4',6-diamidino-2-phenylindole), and images were acquired using a CLSM.

6. Intracellular Trafficking Localization of LPC-Lipo

The intracellular co-localization of LPC-Lipo with $Rab11^+$ recycling endosome and lysosome was assessed using CLSM imaging. The bEnd.3 cells were seeded into a confocal dish and then incubated with DiO-labeled Lipo or LPC-Lipo. Following incubation, the cells were washed twice with PBS. For labeling lysosome, live cells were stained with LysoTracker Red (Beyotime, 50 nM, 30 min) according to manufacturer's protocol. For labeling endosome, cells were fixed with 4% PFA, permeabilized with 0.1% Triton X-100, blocked with 10% FBS. Primary antibodies against

early endosome marker EEA1 (Cell Signaling Technology, #48453, 1:200) or recycling endosome marker Rab11 (Cell Signaling Technology, #5589, 1:100) were applied overnight at 4°C, followed by appropriate Alexa Fluor-conjugated secondary antibodies (1 h, room temperature). For p62 co-localization studies, fixed cells were co-stained with anti-p62 antibody (Proteintech, #18420-1-AP, 1:750) alongside either anti-EEA1 or anti-Rab11 antibodies using the aforementioned protocol. For endosomal density quantification, post-treated cells underwent identical fixation/permeabilization/blocking procedures before incubation with anti-EEA1 or anti-Rab11 antibodies, followed by Alexa Fluor 488 Phalloidin (1:400, cytoskeletal visualization) and DAPI nuclear counterstain. All images were acquired using a CLSM system. Co-localization efficiency and endosomal density were quantified in ImageJ with three representative fields per condition. Pearson correlation coefficient was quantified with ImageJ.

7. Western Blot (WB) Analysis

The expressions levels of EEA1, Rab11 and p62 in bEnd.3 cells were analyzed by western blotting. In brief, total protein was extracted using RIPA Buffer (Solarbio, China) and quantified with a BCA assay kit (Solarbio). Protein lysates were separated by 10% SDS-polyacrylamide gel electrophoresis (SDS-PAGE) and transferred onto polyvinylidene fluoride (PVDF) membranes (Millipore, USA). Membranes were incubated overnight at 4°C with the following primary antibodies: anti-EEA1 (Cell Signaling Technology, #48453, 1:1000), anti-Rab11 (Cell Signaling Technology, #5589, 1:1000), and anti-p62/SQSTM1 (Proteintech, #18420-1-AP, 1:5000). This was followed by incubation with horseradish peroxidase (HRP)-conjugated secondary antibodies (Promega, 1:10,000) for 1 hour at room temperature. β-Actin (Proteintech, #66009-1-lg, 1:5000) served as the loading control. Immunoreactive bands were detected using a gel imaging system

(Protein Simple, USA), and band intensity was quantified by grayscale analysis using ImageJ software.

8. Intracranial PTX Concentration Assay

In an intracranial PDX model, twelve hours after intravenous administration of Lipo(PTX) and LPC-Lipo(PTX), mice were sacrificed and whole brains were collected. PTX concentrations in the ipsilateral (tumor-bearing) and contralateral brain hemispheres were quantified using ultra performance liquid chromatography coupled with triple quadrupole mass spectrometry (UPLC-MS/MS; Waters, USA). Brain tissues were weighed and homogenized in ultrapure water (1:3, w/v) using a cryogenic grinder (60 Hz, 4 min; cycles: 55 s grinding/5 s pause). Homogenates (50 µL) were mixed with 50 µL methanol and 150 µL ice-cold precipitation agent (acetonitrile containing buspirone as internal standard), vortexed vigorously for 1 min, and centrifuged at 12,000 rpm for 10 min. The supernatant was filtered (0.22 µm) and injected (5 µL) into the UPLC-MS/MS system. Chromatographic separation was performed on a C18 column (100 mm × 2.1 mm, 1.7 µm) using an isocratic mobile phase of methanol: water (75:25, v/v) at a flow rate of 0.3 mL/min. PTX concentrations were determined using a validated standard curve.

9. In Vivo Anti-tumor Activity of LPC-Lipo(PTX) in CT2A model

To establish a mouse intracranial GBM model, CT2A cells were implanted into brains of female C57BL/6 mice under the guidance of a stereotactic instrument at coordinates relative to bregma: 2.0 mm posterior, 2.0 mm lateral, and 3.0 mm ventral. Five days post-implantation, tumor-bearing mice were randomized into three groups (n = 5) and treated intravenously via tail vein with: Saline, Lipo(PTX), and LPC-Lipo(PTX) (both 8 mg/kg PTX-equivalent). Treatments were administered every 2 days for five total doses. Tumor progression was assessed by bioluminescence imaging on

days 5, 10, and 17. On day 17, brains were processed for H&E staining as above. Kaplan-Meier survival curves were generated in independent animal cohorts.

10. Flow Cytometry Analysis of Tumor-Infiltrating T Cells

Single-cell suspensions were prepared from CT2A tumors post treatment using enzymatic digestion. For T cell profiling, cells were stained with fluorochrome-conjugated antibodies against mouse surface markers: FITC anti-CD45.2 (BioLegend, #109806), APC anti-CD3 (BioLegend, #100236), PE anti-CD4 (BioLegend, #100408), and PerCP/Cy5.5 anti-CD8a (BioLegend, #100734). After 1-h incubation at 4°C protected from light, cells were washed twice with cold PBS to remove unbound antibodies. Samples were acquired on a BD FACVerse flow cytometer and analyzed using FlowJo software (v10.6.2). Live lymphocytes were gated based on forward/side scatter properties and CD45.2+ expression prior to T cell subset analysis.

11. In Vivo Biosafety and Neurotoxicity Evaluation

For systemic toxicity assessment, blood samples were collected from mice treated with LPC-Lipo or LPC-Lipo(PTX) for analysis of hematological parameters (including WBCs, RBCs, HGB, Gran%, and PLTs) and serum biochemical markers (including ALT, AST, ALP, and TBIL). Major organs (heart, liver, spleen, lungs, and kidneys) were collected and fixed in 4% paraformaldehyde for paraffin embedding and H&E staining.

Neurobehavioral functions were evaluated using the open field test and novel object recognition test. In the open field test, mice were allowed to freely explore an arena (50 × 50 × 40 cm) for 10 min, and their total distance moved and time spent in the center were recorded. For the novel object recognition test, mice were first familiarized with two identical objects, then one object was replaced with a novel one after 1 h. The recognition index was calculated based on exploration time.

12. In Vitro Tumor Treating Fields (TTFields) Assay

TBD0220 and U87 cell suspensions were dropped onto glass coverslips placed in ceramic dishes, and 5 mL of medium was added to cover the slides after the cells adhered. Cells were exposed to tumor-treating fields (TTFields) using an in vitro applicator system under controlled conditions: 200 kHz alternating field, bidirectional perpendicular switching, and maintained field strength of 2.04 V/cm. Environmental parameters were strictly regulated at 31°C, 90% humidity, and 5% CO_2. Five experimental groups were established in triplicate: (1) Untreated control; (2) LPC-Lipo(PTX); (3) TTFields (6 h exposure); (4) TTFields (24 h exposure); (5) LPC-Lipo(PTX) + TTFields (6 h concurrent treatment).

Following treatment, cells underwent comprehensive analysis. Microtubule morphology was assessed by immunostaining with anti-α-tubulin antibody (Proteintech, #11224-1-AP, 1:200) according to established protocols. Cell cycle distribution was determined through propidium iodide staining using the Cell Cycle Analysis Kit (Beyotime, C1052) and analyzed via flow cytometry. Expression of cell cycle regulatory proteins was evaluated via standard western blot procedures, with the following antibodies: anti-p21 (Cell Signaling Technology, #2947, 1:1000), anti-CDK1 (Proteintech, #19532-1-AP, 1:1000), and Cyclin B (Proteintech, #55001-1-AP, 1:1000). Cell proliferation activity was quantified using the BeyoClick™ EdU-594 Assay Kit (Beyotime, C0078) with subsequent confocal microscopy imaging.

13. DNA Damage Analysis

TBD0220 and U87 cells were treated with LPC-Lipo(PTX) and TMZ. DNA damage repair-related protein expression was analyzed by Western blotting using antibodies against γ-H2AX (ABclonal, AP1555, 1:1000), XRCC1 (Proteintech, #21468-1-AP, 1:1000), RAD51 (Proteintech, #14961-1-AP, 1:1000), and MRE11 (Proteintech, #10744-1-AP, 1:1000). The expression level of γ-H2AX

was further assessed via immunofluorescence staining. Apoptosis-related proteins, Caspase-3 and Caspase-7, were detected by WB analysis using their respective specific antibodies (Proteintech, #19677-1-AP and 27155-1-AP, 1:500). Cellular apoptosis rate was quantified using an apoptosis detection kit.

References

1 Qi Y, Ingolfsson HI, Cheng X, Lee J, Marrink SJ, Im W. CHARMM-GUI Martini Maker for Coarse-Grained Simulations with the Martini Force Field. *J Chem Theory Comput* 2015;**11**:4486-94.

2 Souza PCT, Alessandri R, Barnoud J, Thallmair S, Faustino I, Grunewald F, et al. Martini 3: a general purpose force field for coarse-grained molecular dynamics. *Nat Methods* 2021;**18**:382-8.

3 Humphrey W, Dalke A, Schulten K. VMD: visual molecular dynamics. *J Mol Graph* 1996;**14**:33-8, 27-8.
